# Interactions between human milk oligosaccharides, microbiota and immune factors in milk of women with and without mastitis

**DOI:** 10.1038/s41598-022-05250-7

**Published:** 2022-01-25

**Authors:** Irma Castro, Cristina García-Carral, Annalee Furst, Sadaf Khwajazada, Janneiry García, Rebeca Arroyo, Lorena Ruiz, Juan M. Rodríguez, Lars Bode, Leónides Fernández

**Affiliations:** 1 Departament of Nutrition and Food Science, Complutense University of Madrid, Madrid, Spain; 2 Probisearch S.L., Tres Cantos, Spain; 3 Department of Pediatrics and Larsson-Rosenquist Foundation Mother-Milk-Infant Center of Research Excellence, University of California San Diego, La Jolla, CA USA; 4 Department of Microbiology and Biochemistry of Dairy Products, Institute of Dairy Products of Asturias, IPLA-CSIC, Villaviciosa, Spain; 5 Department of Galenic Pharmacy and Food Technology, Complutense University of Madrid, Madrid, Spain

**Keywords:** Applied immunology, Clinical microbiology

## Abstract

Lactational mastitis is an excellent target to study possible interactions between HMOs, immune factors and milk microbiota due to the infectious and inflammatory nature of this condition. In this work, microbiological, immunological and HMO profiles of milk samples from women with (MW) or without (HW) mastitis were compared. Secretor status in women (based on HMO profile) was not associated to mastitis. DFLNH, LNFP II and LSTb concentrations in milk were higher in samples from HW than from MW among Secretor women. Milk from HW was characterized by a low bacterial load (dominated by *Staphylococcus epidermidis* and streptococci), high prevalence of IL10 and IL13, and low sialylated HMO concentration. In contrast, high levels of staphylococci, streptococci, IFNγ and IL12 characterized milk from MW. A comparison between subacute (SAM) and acute (AM) mastitis cases revealed differences related to the etiological agent (*S. epidermidis* in SAM; *Staphylococcus aureus* in AM), milk immunological profile (high content of IL10 and IL13 in SAM and IL2 in AM) and milk HMOs profile (high content of 3FL in SAM and of LNT, LNnT, and LSTc in AM). These results suggest that microbiological, immunological and HMOs profiles of milk are related to mammary health of women.

## Introduction

Human milk is a complex, non-imitable food and is unanimously considered as the best infant feeding option^1^. In addition to its nutritional value, it contains a plethora of bioactive components contributing to infant growth, tissue repair, protection, immune maturation, neurodevelopment, regulation of metabolism, angiogenesis, development of microbial communities, and many others^2,3^. At present, an increasing quantity of human milk oligosaccharides (HMOs), cytokines, chemokines, growth factors, bacterial species and bacterial metabolites have been identified in human milk, and their multiple functions have been described^4–7^. Although most studies have addressed the potential roles of individual HMOs, immune factors and bacterial strains in vitro or animal models, they are present as a diverse and interacting mix in human milk^8,9^. Consequently, combinations of methodologies are required to decipher their individual and collective impact on health.

While studies on human milk bioactive compounds have been focused on their impact in infant health, they too are relevant for mother’s health. Lactational mastitis is one of the most common problems that compromise breastfeeding^10^. Mastitis is not a uniform condition since it can be present as acute, subacute or chronic processes^11–13^. As a consequence, the etiological agents, the course, the symptoms and their severity may vary depending on the cases^11,13^. Because of the inflammatory and infectious nature of the condition, mastitis constitutes an excellent target to study potential interactions among HMOs, soluble immune factors and the milk microbiota. In this context, the objectives of this work were, first, to describe the differences in microbiological, immunological and HMO profiles between milk samples from healthy women and from women with mastitis, and second, to explore possible associations between microbiota, immune compounds and HMO profile in milk samples from cases of acute and subacute mastitis.

## Results

### Comparison of the HMOs profiles in the milk samples from the HW and MW groups

HMO profiles of milk samples from women with mastitis (MW; n = 69) and healthy women (HW; n = 41) are shown in Supplementary Fig. 1. A total of 19 individual HMOs were found in all samples but in different concentrations (Supplementary Fig. 1). In a distinct group of women classified as Secretor (n = 89, 81%), 2′-fucosyllactose (2′FL) was the predominant HMO with a median [IQR] value of 6444.10 [4820.80–7929.50] nmol/mL, meanwhile in non-Secretor women (n = 21, 19%), the highest concentrations corresponded to 3-fucosyllactose (3FL) and lacto-*N*-fucopentaose II (LNFP II). The odds ratio (OR) of the occurrence of mastitis in Secretor women was 1.70 compared to non-Secretor women, but this association was not statistically significant as indicated by the 95% CI of the OR (Table 1).

**Table 1:** Incidence of mastitis and type of mastitis (acute [AM] or subacute [SAM]) among participant women according to the Secretor status.

	Mastitis n (%)	Healthy n (%)	Odds ratio (95% confidence interval)
Secretor	58 (65)	31 (35)	1.70 (0.65–4.45)
Non secretor	11 (52)	10 (48)	

	AM n (%)	SAM n (%)	Odds ratio (95% confidence interval)
Secretor	31 (69)	27 (31)	3.06 (0.73–12.71)
Non secretor	8 (73)	3 (27)	

When comparing MW and HW within the Secretor status, differences in the concentrations of several HMOs were observed (Table 2). The concentrations of DFLNH, LNFP II and LSTb were significantly higher (Wilcoxon sum rank tests; *p* < 0.024) in samples from the HW group, while the content of DSLNT, LNFP I, LNFP III, LNH, LSTc, and 6’SL was higher in samples from the MW group (Wilcoxon rank sum tests; *p* < 0.034). Overall, samples from Secretor MW had about double the concentration of the sialylated, Type 2 (LNnT + LNFP III + LSTc) and α-2,6 (LSTc + 6′SL) HMOs when compared with samples from Secretor samples from HW participants (Supplementary Table 1). Among non-Secretor women, no differences were observed in the concentration of HMOs according to the breast health status (Table 2, Supplementary Table 1).

**Table 2:** Concentration (nmol/mL) of individual HMOs in milk samples according to the Secretor and breast health status (HW, healthy women; MW, mastitis cases) in women.

HMO	Non-secretor		*p-* value^a^	Secretor		*p-*value^a^
	HW (n = 10)	MW (n = 11)		HW (n = 31)	MW (n = 62)	
	Median (IQR) or Mean [95% CI]	Median (IQR) or Mean [95% CI]		Median (IQR) or Mean [95% CI]	Median (IQR) or Mean [95% CI]	
**Fucosylated**						
2′FL	5.05 (2.35–9.35)	16.10 (9.60–34.10)	0.605	6100.98 [649.12]	6576.35 [657.18]	0.757
3FL	5853.60 (5123.90–6865.02)	4748.20 (4314.10–5572.10)	0.605	2206.70 (1517.95–3189.50)	1703.95 (1273.95–2774.47)	0.178
DFLac	1.15 (0.45–5.15)	1.20 (0.65–10.95)	0.919	356.60 (272.25–455.10)	364.45 (288.72–513.75)	0.516
LNFP I	217.37 [61.26]	219.74 [66.23]	0.985	894.50 (482.40–1383.45)	1358.85 (809.07–1954.80)	0.034*
LNFP II	2579.35 [648.66]	2352.17 [723.90]	0.769	1175.20 (801.40–1502.80)	763.15 (481.02–974.82)	0.000*
LNFP III	5.65 (4.07–10.82)	6.50 (4.85–14.20)	0.769	12.30 (8.50–17.00)	22.35 (12.52–34.85)	0.000*
DFLNT	609.86 [214.08]	640.08 [251.10]	0.919	1255.62 [221.55]	1119.90 [162.63]	0.960
FLNH	318.50 (152.32–420.75)	242.80 (169.20–269.90)	0.663	206.10 (118.90–327.20)	181.00 (99.90–289.36)	0.516
DFLNH	516.82 [170.13]	385.74 [161.53]	0.663	232.80 (153.10–282.50)	90.55 (44.65–150.12)	0.000*
**Sialylated**						
3′SL	293.55 (278.12–314.45)	226.60 (141.65–315.15)	0.605	347.10 (292.65–482.85)	331.15 (265.50–438.47)	0.516
6′SL	415.80 (250.72–582.27)	0.605	0.152	328.80 (226.40–547.35)	724.75 (444.85–871.77)	0.000*
LSTb	122.15 [38.01]	98.26 [32.59]	0.663	74.90 (55.95–126.85)	57.85 (42.80–85.50)	0.024*
LSTc	57.25 (30.50–75.27)	134.90 (66.60–194.40)	0.605	79.90 (44.05–101.65)	234.25 (90.32–376.02)	0.000*
DSLNT	13.05 (8.40–27.43)	16.00 (7.65–61.00)	0.919	66.10 (49.55–103.80)	156.32 (73.12–246.60)	0.000*
FDSLNH	130.84 [49.22]	147.31 [56.91]	0.769	85.40 (54.70–124.40)	132.30 (70.90–208.52)	0.154
DSLNH	40.90 [11.93]	49.25 [19.06]	0.919	57.10 (38.55–83.55)	62.10 (40.62–97.77)	0.411
**Neutral**						
LNnT	131.20 (97.35–197.77)	140.60 (103.05–180.35)	0.969	324.10 (235.25–391.95)	350.85 (233.60–474.87)	0.411
LNT	2258.69 [841.13]	1595.31 [539.52]	0.605	1293.90 (1013.40–1727.70)	1223.40 (775.97–1540.47)	0.411
LNH	58.84 [24.29]	89.93 [32.05]	0.605	32.80 (20.65–41.60)	53.15 (33.35–90.75)	0.005*

*CI* confidence interval, *IQR* Interquartile range, *DFLac* difucosyllactose, *DFLNH* difucosyllacto-*N*-hexaose, *DFLNT* difucosyllacto-*N*-tetrose, *DSLNH* diasilyllacto-*N*-hexaose, *DSLNT* diasilyllacto-*N*-tetraose, *FDSLNH* fucodisialyllacto-*N*-hexaose, *FLNH* fucosyllacto-*N*-hexaose, *HMO* human milk oligosaccharide, *LNFP* lacto-*N*-fucopentaose, *LNH* lacto-*N*-hexaose, *LNnT* lacto-*N*-neotetraose, *LNT* lacto-*N*-tetraose, *LSTb* sialyl-lacto-*N*-tetraose b, *LSTc* sialyl-lacto-*N*-tetraose c, *2’FL* 2’-fucosyllactose, *3FL* 3-fucosyllactose, *3’SL* 3’-sialyllactose, *6’SL* 6’-sialyllactose.

*Statistically significant difference, *p* < 0.05.

^a^Wilcoxon rank sum tests or ANOVA tests (depending on the distribution of the data) were used to determine differences in HMOs concentration between samples from mastitis-suffering or healthy women from Secretor or non-Secretor status. FDR-adjusted *p-*values.

**Figure 1:**
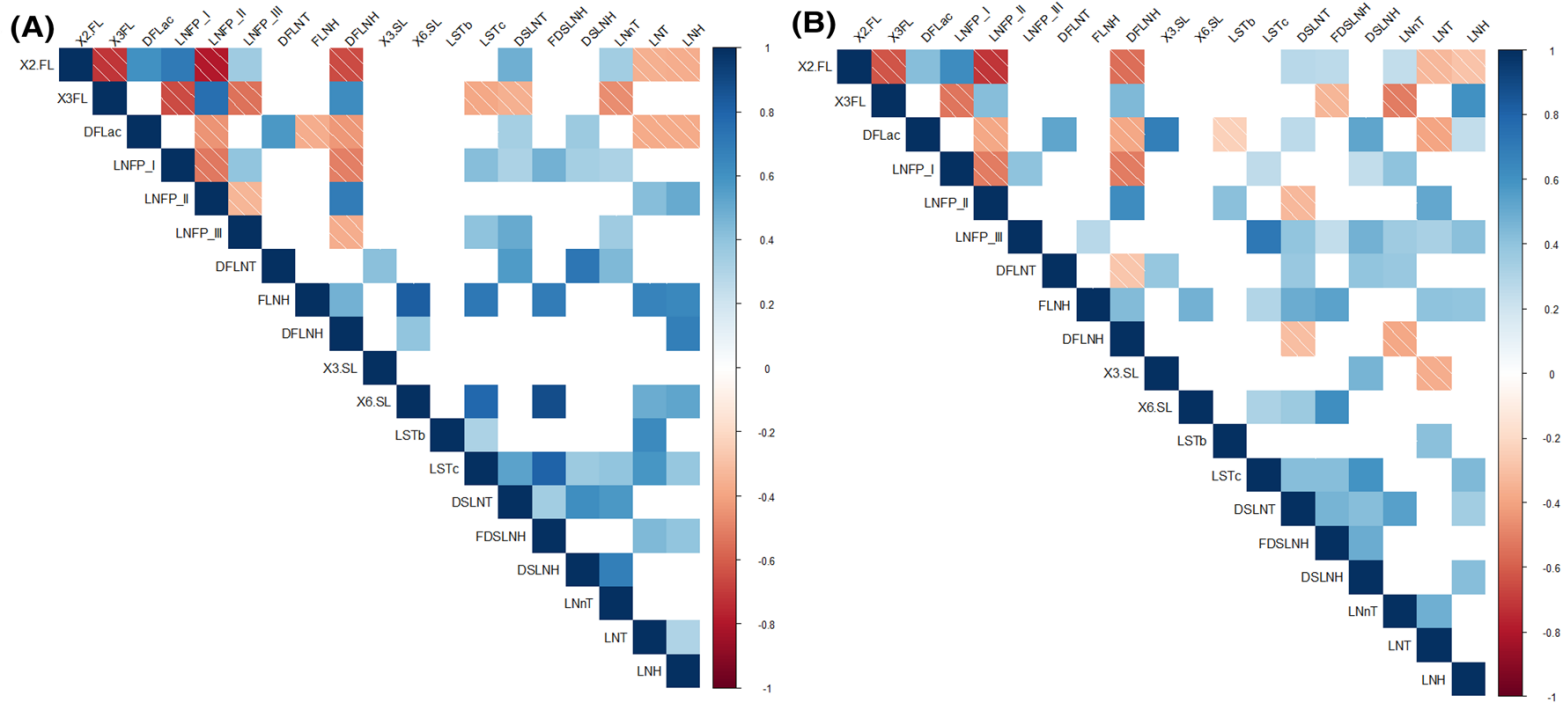
Spearman rank correlations between individual HMOs concentrations in milk samples from healthy women (HW group; **A**) and mastitis cases (MW group; **B**) groups. Only statistically significant (*p* < 0.05) correlations between individual HMOs that were present in > 50% of the samples in each group are shown .The strength and colors indicate directionally (blue denotes positive; red denotes negative) of the association. DFLac, difucosyllactose; DFLNH, difucosyllacto-*N*-hexaose; DFLNT, difucosyllacto-*N*-tetrose; DSLNH, diasilyllacto-*N*-hexaose; DSLNT, diasilyllacto-*N*-tetraose; FDSLNH, fucodisialyllacto-*N*-hexaose; FLNH, fucosyllacto-*N*-hexaose; HMO, human milk oligosaccharide; LNFP, lacto-*N*-fucopentaose; LNH, lacto-*N*-hexaose; LNnT, lacto-*N*-neotetraose; LNT, lacto-*N*-tetraose; LSTb, sialyl-lacto-*N*-tetraose b; LSTc, sialyl-lacto-*N*-tetraose c; 2’FL, 2’-fucosyllactose; 3FL, 3-fucosyllactose; 3’SL, 3’-sialyllactose; 6’SL, 6’-sialyllactose.

Both strong positive and negative correlations, which globally were highly similar in samples from HW and MW groups, were found among the concentrations of some individual HMOs (Fig. 1). In the HW group, the strongest positive correlations were found between 6′SL and LSTc, FLNH, and FDSLNH and between 3FL and LNFP II (Spearman’s ρ > 0.75; *p* = 0.000), while the strongest negative correlations were established between 2’FL and LNFP II and 3FL (Spearman’s ρ ≥ ‒0.72; *p* = 0.000). The intensity of these correlations was lower in samples from the MW group, where the strongest correlations were noted between LSTc and LNFP III (Spearman’s ρ = 0.72; *p* = 0.000) and 2’FL and LNFP II (Spearman’s ρ = ‒0.72; *p* = 0.000) (Fig. 1).

### Comparison of the microbiological profile of milk samples from the HW and MW groups

Milk cultures were performed on all the milk samples (n = 110) and bacterial growth was observed in 106 (97%) samples (Table 3). In contrast, yeasts could only be detected in less than 10% of the samples in both groups. The 4 samples where bacterial growth was not detected had been collected from the HW group. The total bacterial counts in the samples where growth was observed from the HW group (n = 37) ranged from 1 to 5.89 log_10_ CFU/mL of milk and the median (IQR) value was 2.67 (1.60–3.18) log_10_ CFU/mL. In contrast, microbial counts in the samples from the MW group (n = 69) ranged from 4.69 to 6.19 log_10_ CFU/mL and had a median (IQR) value of 5.31 (5.03–5.53) log_10_ CFU/mL. Therefore, the microbial counts in the MW group samples were more than 100-fold higher than in the HW group samples (Wilcoxon rank sum test; *p* = 0.000) (Table 3).

**Table 3:** Microbiological counts (log_10_ CFU/mL) using culture-dependent analysis of milk samples from healthy women (HW) and mastitis cases (MW) where growth was detected (n = 106).

Microorganism	HW (n = 37)		MW (n = 69)		*p-*value^c^	*p-*value^d^
	n (%)^a^	Median (IQR)^b^	n (%)	Median (IQR)		
**Firmicutes**						
*Staphylococcus epidermidis*	35 (95)	2.50 (1.74–2.81)	59 (85)	5.00 (4.52–5.30)	*0.454*	0.000*
*Staphylococcus aureus*	3 (8)	1.30 (1.18–1.70)	39 (52)	4.87 (3.84–5.30)	0.000*	0.007*
Other staphylococci^e^	5 (13)	2.30 (2.00–2.84)	8 (12)	3.80 (3.02–4.66)	*1.000*	0.027*
*Streptococcus mitis/oralis*	9 (24)	1.84 (1.00–2.46)	19 (27)	4.18 (3.42–5.04)	1.000	0.000*
*Streptococcus salivarius*	4 (11)	1.93 (1.74–2.24)	18 (26)	4.18 (3.70–4.70)	0.300	0.005*
Other streptococci^f^	13 (35)	1.90 (1.30–2.08)	12 (17)	3.80 (3.02–4.66)	0.315	0.000*
*Enterococcus* sp.	2 (5)	3.53 (3.23–3.71)	3 (4)	4.18 (4.03–4.76)	*1.000*	0.433
Other Firmicutes^g^	7 (19)	2.30 (2.04–3.06)	6 (9)	4.18 (3.84–4.38)	*0.454*	0.008*
**Proteobacteria**^h^	3 (8)	3.60 (3.31–3.73)	5 (7)	2.60 (2.54–5.10)	*1.000*	0.881
**Actinobacteria**						
*Rothia mucilaginosa*	4 (11)	1.78 (1.68–1.81)	9 (13)	3.78 (3.44–4.00)	*1.000*	0.032*
*Corynebacterium* sp.	5 (13)	1.00 (1.00–1.30)	9 (13)	3.70 (3.39–4.18)	*1.000*	0.006*
Other Actinobacteria^i^	9 (24)	2.36 (1.30–3.00)	0 (0)	–	0.000*	
**Yeast**	2 (5)	2.02 (1.76–2.18)	4 (6)	3.14 (2.84–4.15)	*1.000*	0.157
**Not identified**	20 (54)	2.41 (2.03–2.94)	0 (0)	–	*0.000	–
**Total CFU**	37 (100)	2.67 (1.60–3.18)	69 (100)	5.31 (5.03–5.53)	*1.000*	*0.000

*IQR* interquartile range, *CFU* colony-forming units.

*Statistically significant difference, *p* < 0.05.

^a^n (%): number (percentage) of samples in which the microorganism was detected (relative frequency of detection).

^b^All data expressed as median (IQR) log_10_ CFU/mL (only samples where bacterial growth was detected).

^c^χ^2^ or Fisher tests (in italics) were used to determine a possible association between breast health status and individual microorganisms or group of microorganisms isolated from milk samples. FDR-adjusted *p*-values.

^d^Wilcoxon rank sum tests were used to determine if there were differences in microbiological counts between samples from HW and MW groups. FDR-adjusted *p*-values.

^e^Other staphylococcal species that were identified include *S. hominis*, *S. lugdunensis, S. pasteuri* and *S. warneri.*

^f^Other streptococcal species that were identified include *S. anginosus*, *S. gordonii, S. parasanguinis, S. pneumoniae* and *S. vestibularis.*

^g^Other Firmicutes includes *Bacillus*, *Lactococcus*, former *Lactobacillus* and *Weisella.*

^h^Proteobacteria includes *Brevundimonas*, *Enterobacteriaceae, Moraxella, Rhizobium* and *Stenotrophomonas.*

^i^Other Actinobacteria includes *Actinomyces, Bifidobacterium*, *Cutibacterium*, *Kocuria* and *Propionibacterium.*

*Staphylococcus epidermidis* was the most frequently isolated species in both groups of samples being detected in 95% of samples from the HW group and in 85% of the samples from the MW group (Table 3). However, a large difference was observed in the levels recorded in both groups. *S. epidermidis* median (IQR) count was 2.50 (1.74–2.81) log_10_ CFU/mL in samples from the HW group but it was double (5.00 [4.52–5.30] log_10_ CFU/mL) in samples from the MW group. *Staphylococcus aureus* was the second species more frequently isolated (52%) and more abundant (median [IQR] count of 4.87 [3.84–5.30] log_10_ CFU/mL) in the MW group (Table 3). Differences were found both in *S. aureus* prevalence (χ^2^ test; *p* = 0.000) and colony counts (Wilcoxon rank sum test; *p* = 0.007) between samples from HW and MW groups. *Streptococcus mitis/oralis* (the MALDI-TOF technique does not allow the differentiation between these two closely related species) and *Streptococcus salivarius* were also detected in significantly higher colony counts in samples from the MW group than in those from the HW group (Wilcoxon rank sum tests; *p* < 0.005), but their prevalences were similar in both groups (Table 3). Other bacterial species and taxonomic groups were found in both groups (HW and MW) of samples except for “Other Actinobacteria (*Actinomyces*, *Bifidobacterium*, *Cutibacterium* and *Kocuria*)” and “Not identified” groups that were detected only in samples from the HW group (Table 3). On the other hand, colony counts of other bacterial species and groups, including other staphylococci, other streptococci, other Firmicutes, *Rothia mucilaginosa* and *Corynebacterium* sp., were higher in the MW group samples than in the HW group samples (Table 3).

Globally, the bacterial counts of the different microbial groups did not differ between the non-Secretor and Secretor samples of HW and MW groups (Supplementary Table 2). A trend could be observed in the counts of *S. epidermidis* and the median total microbial counts, which were about 0.70 log_10_ CFU/mL higher in non-Secretor samples from the HW than in samples from the Secretor HW group (Supplementary Table 2). No differences in microbiological counts were found in the set of samples from the MW group (Supplementary Table 2). The comparison of the prevalence of individual bacterial species or groups according to the Secretor and breast health status of the women indicated that there was no association between both factors (Fisher tests; *p* > 0.05) (Supplementary Table 2).

### Comparison of the immunological profile of milk samples from the HW and MW groups

The immunological profile including 18 immune compounds was characterized in 95 samples (41 samples from the HW group and 54 samples from the MW group) (Table 4). All compounds were detected in, at least, one sample of the whole set of analysed samples, although at different prevalence and abundance. In particular, notable variations were registered when comparing samples from the HW and MW groups (Table 4). Regarding innate immunity compounds, IL1β, IL6, IFNγ and TNFα were detected in more samples in the MW group (in 78% of the samples) than in the HW group (in 56% of the samples) (χ^2^ tests; *p* ≤ 0.000). In addition, the concentrations (expressed as median [IQR]) of IL1β and TNFα were also higher in samples from the MW group (8.10 [1.16–23.41] ng/L for IL1β and 29.02 [12.81–72.70] ng/L for TNFα) than in samples from the HW group (1.10 [0.33–2.20] ng/L for IL1β and 3.15 [2.02–4.52] ng/L for TNFα) (Wilcoxon rank sum tests; *p* ≤ 0.003). In contrast, no differences were observed in IL6 and INFγ concentrations between both groups of samples (Table 4).

**Table 4:** Frequency and concentration of immune factors detected in milk samples from healthy women (HW) and mastitis cases (MW).

	HW (n = 41)		MW (n = 54)		*p*-value^b^	*p*-value^c^
	n (%)^a^	Median (IQR)	n (%)	Median (IQR)		
**Innate immunity**						
IL1β (ng/L)	23 (56)	1.10 (0.33–2.20)	51 (94)	8.10 (1.16–23.41)	0.000*	0.003*
IL6 (ng/L)	17 (41)	12.70 (6.80–18.40)	42 (78)	10.60 (2.42–36.81)	0.000*	0.684
IL12(p70) (ng/L)	6 (14)	0.55 (0.12–0.90)	8 (15)	2.29 (0.76–4.69)	0.796	0.331
IFNγ (ng/L)	1 (2)	4.70	52 (96)	54.43 (11.22–259.87)	0.000*	–
TNFα (ng/L)	18 (44)	3.15 (2.02–4.52)	54 (100)	29.02 (12.81–72.70)	0.000*	0.000*
**Acquired immunity**						
IL2 (ng/L)	0 (0)	-	38 (70)	6.55 (0.98–18.68)	0.000*	–
IL4 (ng/L)	1 (2)	0.70	28 (52)	0.65 (0.26–1.39)	0.000*	–
IL10 (ng/L)	33 (80)	3.30 (1.75–4.32)	6 (11)	3.51 (3.29–4.40)	0.000*	0.680
IL13 (ng/L)	33 (80)	2.70 (1.60–3.90)	10 (18)	0.76 (0.35–1.24)	0.000*	0.000*
IL17 (ng/L)	3 (7)	4.30 (3.20–5.40)	31 (57)	18.30 (8.54–51.43)	0.000*	0.227
**Chemokines**						
IL8 (ng/L)	41 (100)	72.10 (32.10–182.30)	54 (100)	270.94 (120.96–1818.12)	*1.000*	0.000*
MCP1 (ng/L)	32 (78)	156.55 (71.17–283.30)	53 (98)	380.45 (80.27–1040.27)	0.003*	0.012*
MIP1β (ng/L)	39 (95)	30.70 (16.60–68.15)	54 (100)	25.34 (9.75–161.30)	*0.092*	0.922
**Hematopoyetic factors**						
IL5 (ng/L)	3 (7)	2.60 (1.70–2.70)	15 (28)	33.30 (18.16–59.57)	0.015*	0.019*
IL7 (ng/L)	38 (93)	34.60 (27.27–53.32)	22 (41)	22.41 (11.46–62.17)	0.000*	0.313
GCSF (ng/L)	29 (71)	18.30 (4.90–57.40)	51 (94)	201.40 (51.06–696.52)	0.000*	0.000*
GMCSF (ng/L)	3 (7)	12.90 (6.65–18.35)	19 (35)	4.17 (1.31–5.14)	0.005*	0.398
TGFβ_2_ (µg/L)	41 (100)	2.00 (1.10–3.40)	54 (100)	1.34 (0.45–6.25)	*1.000*	0.471

*GCSF* granulocyte colony-stimulating factor, *GMCSF* granulocyte–macrophage colony-stimulating factor, *INFγ* interferon-γ, *IL* interleukin, *MCP1* macrophage-monocyte chemoattractant protein-1, *MIP1β* macrophage inflammatory protein-1β, *TGFβ*_*2*_ transforming growth factor-β_2_, *TNFα* tumor necrosis factor-α.

*Statistically significant difference, *p* < 0.05.

^a^n (%): number (percentage) of samples in which the immunological compound was detected (relative frequency of detection).

^b^χ^2^ or Fisher tests (in italics) were used to determine a possible association between the breast health status and the immunological compound. FDR-adjusted *p*-values.

^c^Wilcoxon rank sum tests were used to determine differences in the concentration detected of each immunological compound between samples from the HW and MW groups. FDR-adjusted *p*-values.

All compounds related to acquired immunity also occurred at different prevalence in samples from mastitis and healthy women (Table 4). IL2, IL4 and IL17 were found more frequently in samples from the MW group than in the HW group, while the opposite was noted for IL10 and IL13 (χ^2^ tests; *p* = 0.000). However, similar concentrations of these compounds related to acquired immunity (IL2, IL4, IL10, and IL17) were found in samples from both groups, with the exception of IL13. The concentration of IL13 (expressed as median [IQR]) was almost four times higher in the HW group samples than in the MW group samples (2.70 [1.60–3.90] ng/L and 0.76 [0.35–1.24] ng/L, respectively; Wilcoxon rank sum test; *p* = 0.000).

Chemokines IL8, MCP1 and MIP1β were detected in a high percentage of samples, although MCP1 was less frequently detected in samples from the HW group than in those from the MW group (Fisher’s exact test; *p* = 0.003) (Table 4). Overall, chemokines were the most abundant immune compounds in milk samples after TGFβ_2_. MCP1 and IL8 concentrations were double- and triple-fold higher, respectively, in samples from the MW group than in samples from the HW group (Wilcoxon rank sum tests; *p* = 0.012 and *p* = 0.000, respectively).

Finally, the hematopoietic factor TGFβ_2_, the most abundant immune factor in milk, was detected in all samples and at similar content (Table 4). In contrast, IL5 and GCSF were more frequently detected (χ^2^ tests; *p* < 0.015) and their levels were approximately tenfold higher (Wilcoxon rank sum tests; *p* < 0.019) in samples from the MW group than in those from the HW group. GMCSF was more prevalent in samples from the MW group, whereas on the contrary IL7 prevailed in samples from healthy participants (χ^2^ tests; *p* < 0.005).

When comparing the prevalence and concentration of this set of immunological compounds among Secretor or non-Secretor women in both groups, there were differences only in the group of healthy women (Supplementary Table 3). The prevalence of IL1β and GCSF and the concentration of IL13 and IL7 were higher in non-Secretor compared to Secretor women in the HW group (Supplementary Table 3).

**Figure 2:**
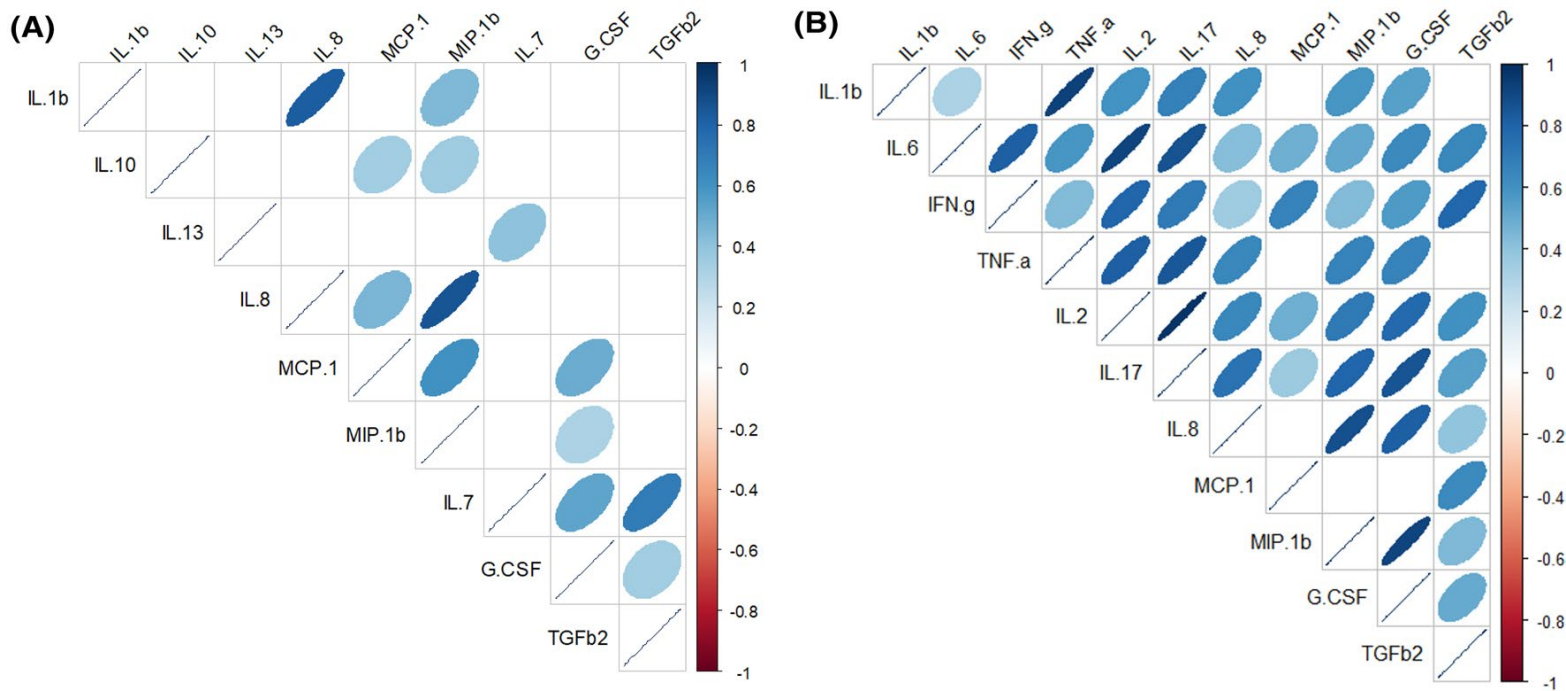
Spearman rank correlations between immunological compounds concentrations in milk samples from healthy women (HW group; **A**) and mastitis cases (MW group; **B**). Only statistically significant (*p* < 0.05) correlations between individual HMOs that were present in > 50% of the samples in each group are shown. The strength and colors indicate directionally (blue denotes positive; red denotes negative) of the association. G.CSF, granulocyte colony-stimulating factor; INF.g, interferon-γ; IL, interleukin; MCP.1, macrophage-monocyte chemoattractant protein-1; MIP1.b, macrophage inflammatory protein-1β; TGFb2, transforming growth factor-β_2_; TNF.a, tumor necrosis factor-α.

Across all samples from the MW group, strong positive correlations were registered between most of the immunological compounds, especially emphasizing those between IL17 and IL2 (Spearman’s ρ = 0.97), TNFα and IL1β (Spearman’s ρ = 0.93), IL6 and IL2 (ρ = 0.92), and MIP1β and GCSF (Spearman’s ρ = 0.91) (Fig. 2). On the contrary, on the set of samples from the HW group, the correlations were weaker and the pairs MIP1β and IL8 (Spearman’s ρ = 0.85) and IL1β and IL8 (Spearman’s ρ = 0.82) registered the strongest correlations (Fig. 2).

### Global comparison of HMO profiles with immunological and microbial profiles in milk samples from the HW and MW groups

**Figure 3:**
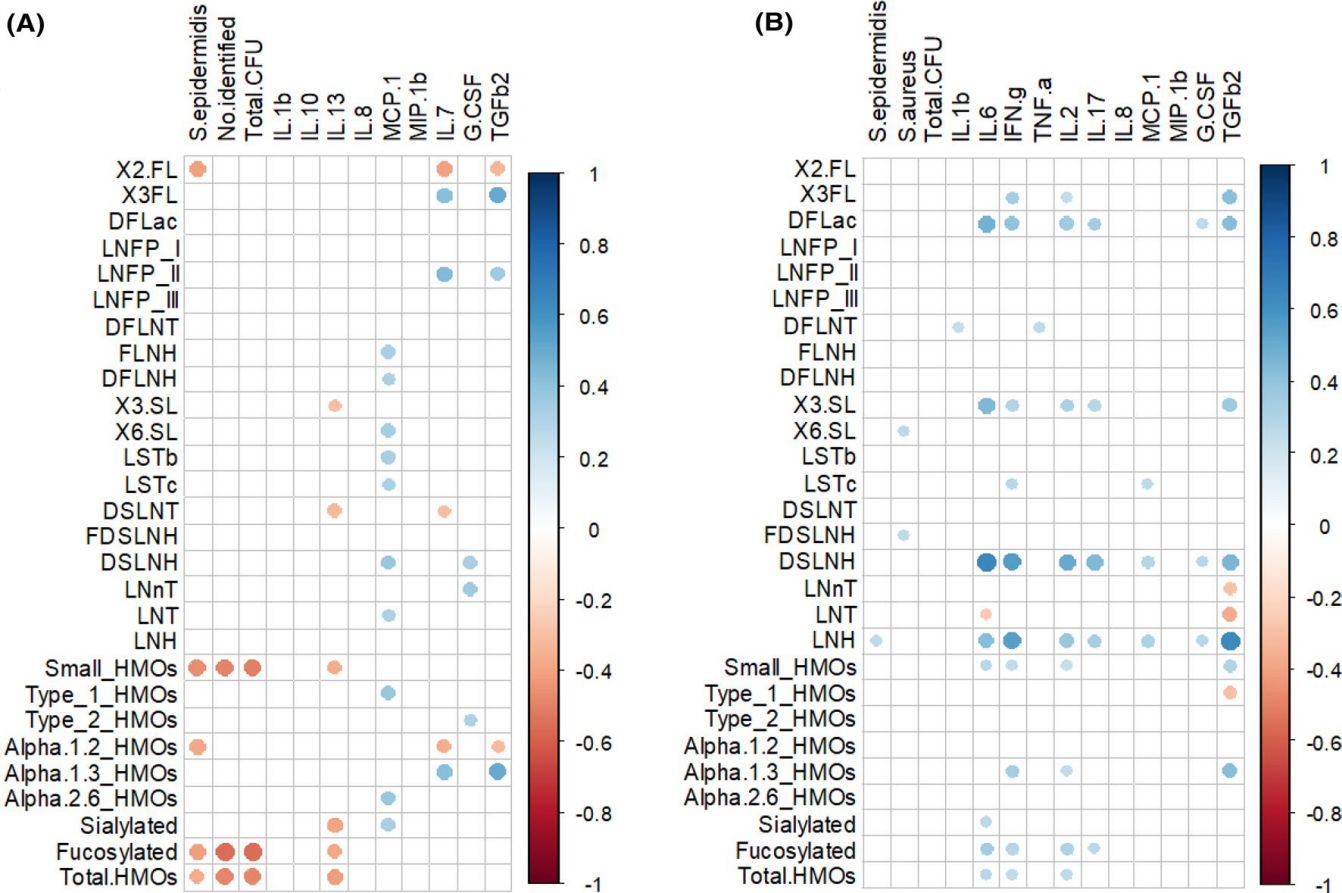
Spearman rank correlations between individual and grouped HMOs, microbial counts and immunological compounds concentrations in milk samples from healthy women (HW group; **A**) and mastitis cases (MW group; **B**). Only statistically significant (*p* < 0.05) correlations between those individual or grouped compounds or bacteria that were present in > 50% of the samples in each group are shown. The strength and colors indicate directionally (blue denotes positive; red denotes negative) of the association. DFLac, difucosyllactose; DFLNH, difucosyllacto-*N*-hexaose; DFLNT, difucosyllacto-*N*-tetrose; DSLNH, diasilyllacto-*N*-hexaose; DSLNT, diasilyllacto-*N*-tetraose; FDSLNH, fucodisialyllacto-*N*-hexaose; FLNH, fucosyllacto-*N*-hexaose; HMO, human milk oligosaccharide; LNFP, lacto-*N*-fucopentaose; LNH, lacto-*N*-hexaose; LNnT, lacto-*N*-neotetraose; LNT, lacto-*N*-tetraose; LSTb, sialyl-lacto-*N*-tetraose b; LSTc, sialyl-lacto-*N*-tetraose c; 2’FL, 2’-fucosyllactose; 3FL, 3-fucosyllactose; 3’SL, 3’-sialyllactose; 6’SL, 6’-sialyllactose. Total HMO-bound sialic acid; total HMO-bound fucose; small HMO; type 1; type 2; α-1,2; α-1,3; and α-2,6 were calculated as the sum of all sialic acid moieties bound to each HMO; all fucose moieties bound to each HMO; 2’FL + 3FL + 3’SL + 6’SL; LNT + LNFP I + LNFP II + LSTb + DSLNT; LNnT + LNFP III + LSTc; LNFP I + 2’FL; LNFP III + 3FL; and LSTb + LSTc + 6’SL, respectively. GCSF, granulocyte colony-stimulating factor; INFγ, interferon-γ; IL, interleukin; MCP1, macrophage-monocyte chemoattractant protein-1; MIP1β, macrophage inflammatory protein-1β; TGFβ_2_, transforming growth factor-β_2_; TNFα, tumor necrosis factor-α.

The levels of *S. epidermidis* and “No identified” microorganisms were negatively correlated with the concentration of small and fucosylated HMOs (Spearman’s ρ ≥ ‒0.41; *p* = 0.000) in samples from the HW group but not in those from the MW group (Fig. 3). On the other hand, in the MW group, *S. aureus* was positively correlated to 6’SL and FDSLNH (Spearman’s ρ = 0.25–0.26; *p* = 0.000) while *S. epidermidis* did to LHN (Spearman’s ρ = 0.26; *p* = 0.000).

In the HW group, the content of two of the most abundant HMOs, 3FL and LNFP II, were positively correlated to the most abundant immune compound in human milk, TFGβ2, and to IL7 (Spearman’s ρ = 0.36—0.50) (Fig. 3). In contrast, TFGβ2, and IL7 were negatively correlated to 2’FL (Spearman’s ρ = − 0.33 to − 0.41). In this group of samples, IL13 showed negative correlation with sialylated HMOs (3’SL and DSLNT) while the opposite was observed for MCP1, which was positively correlated to several HMOs (FLNH, DFLNH, 6’SL, LSTb, LSTc, DSLNH and, LNT). A different pattern of correlations were noted in samples from the MW group since the strongest positive correlations were those established between the pairs TFGβ2/LNH and IL6/DSLNH (Spearman’s ρ = 0.64). In this group, the strongest negative correlation was between TFGβ2 and LNT and LNnT. Globally, in samples from the MW group, IL17, IL2, IL6, INFγ and TFGβ2 were positively correlated to some HMOs including 3FL, DFLac, 3’SL, DSLNH and LNH (Fig. 3).

In order to visualize the global differences in the characteristics of all the samples, a heatmap was performed taking into account the content of HMOs, immune factors and viable bacteria that were present in at least 20% of the samples (Supplementary Fig. 2). In accordance with some results that have already been presented above, the main factor that determines the clustering of the samples was the breast health status (healthy *vs.* mastitis), and this clustering is mainly related to the bacterial load and the composition and profile of immunological factors. Samples from the MW group were characterized by a high load of *S. epidermidis*, *S. aureus*, *S. salivarius*, *S. mitis/oralis* and/or other streptococci, a higher concentration of IFNγ and IL2 and a lower content of IL7, IL10 and IL13. In contrast, the microbial groups “Other Actinobacteria” and “No identified” were only found in samples from healthy women, usually accompanied by low level of *S. epidermidis* and different species of streptococci and an immunological profile almost opposite to samples from the MW group (higher prevalence of IL7, IL10 and IL13) (Fig. 3). However, one of the samples from the MW group was similar to samples from the HW group according to all the characteristics used for this clustering. The HMO profiles of the milk samples did not seem to have an important contribution to this clustering as samples with Secretor and non-Secretor status were more or less equally distributed among healthy and mastitis women (Supplementary Fig. 2).

### Comparison of HMO and immunological profiles in milk samples from acute and subacute mastitis suffering women

In 29 samples out of 69 from mastitis suffering women, which corresponded to AM cases, *S. aureus* was detected at a particularly high rate (mean [IQR] = 4.88 [3.85–5.30] log_10_ CFU/mL) (Supplementary Fig. 3). In the rest of samples (n = 40), which corresponded to SAM cases, *S. aureus* was not detected or detected at a very low concentration (Wilcoxon rank sum test; *p* = 0.000). In addition, the prevalence of *S. aureus* was also higher in samples from AM than in those from SAM cases, as it was present in all samples from AM cases but only in 25% of samples from SAM cases and at lower concentration (mean [IQR] = 3.06 [2.80–3.42] log_10_ CFU/mL) (χ^2^ test; *p* = 0.000).

There were no more differences between samples from AM and SAM cases in relation to total bacterial counts or to the levels of other specific bacteria (Supplementary Table 4, Supplementary Fig. 3). There was not association between the type of mastitis and the Secretor status (OR [95% CI] = 3.06 (0.73–12.71) (Table 1).

**Figure 4:**
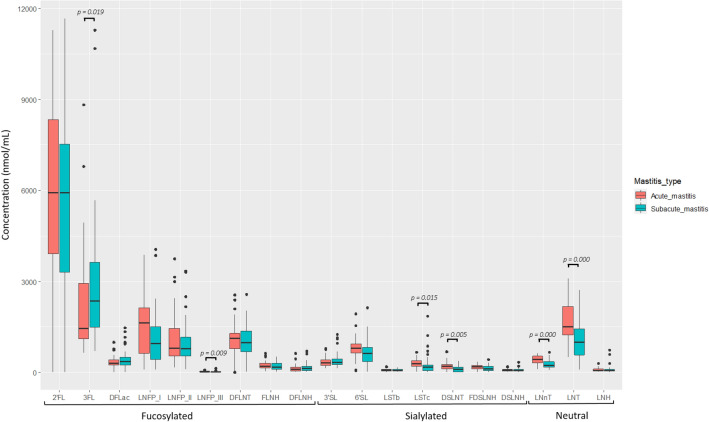
Box-plots showing the concentrations (nmol/mL) of the individual HMOs. The boxes represent the values of the interquartile ranges, with the median represented as a line. Outliers are represented as dots. Samples colored in red belong to acute mastitis (AM) cases and those in green to subacute mastitis (SAM) cases. Wilcoxon rank sum tests or ANOVA tests (depending on the distribution of the data) were used to determine differences in HMOs concentration between samples from acute or subacute mastitis-suffering women. DFLac, difucosyllactose; DFLNH, difucosyllacto-*N*-hexaose; DFLNT, difucosyllacto-*N*-tetrose; DSLNH, diasilyllacto-*N*-hexaose; DSLNT, diasilyllacto-*N*-tetraose; FDSLNH, fucodisialyllacto-*N*-hexaose; FLNH, fucosyllacto-*N*-hexaose; HMO, human milk oligosaccharide; LNFP, lacto-*N*-fucopentaose; LNH, lacto-*N*-hexaose; LNnT, lacto-*N*-neotetraose; LNT, lacto-*N*-tetraose; LSTb, sialyl-lacto-*N*-tetraose b; LSTc, sialyl-lacto-*N*-tetraose c; 2’FL, 2’-fucosyllactose; 3FL, 3-fucosyllactose; 3’SL, 3’-sialyllactose; 6’SL, 6’-sialyllactose.

According to the type of mastitis, AM or SAM, statistically significant differences were observed in the concentration of the individual HMOs LNFP III, DSLNT, LNnT and LNT as well as of Type 1 and Type 2 HMOs (Fig. 4, Supplementary Fig. 4, Supplementary Tables 5, 6). The content of all these HMOs were higher among samples from AM cases than in samples from SAM cases (Wilcoxon rank sum tests; *p* ≤ 0.042).

Regarding the immunological profile, IL2 was found in most of the samples from the AM group, whereas IL10 and IL13 were detected more frequently in samples from the SAM group (Supplementary Table 7). Differences in the concentration were only found for IL5 whose concentration in the samples from the SAM cases was about five-fold of that found in AM cases (Supplementary Table 7).

## Discussion

Breast pain associated to mastitis is a leading cause of breastfeeding discontinuation. Because breastmilk is considered the optimal infant nutrition, mastitis may deprive infants of its multiple benefits during this critical development stage. Therefore, mastitis should be considered a relevant public health issue. This study reports notable differences in the microbiological, immunological, and HMO profiles of milk samples from lactating mothers with mastitis and healthy controls. In addition, it describes the main microbiological features of milk samples from AM and SAM cases, and reveals trends in immune and HMO profiles which may be useful to assess the risk of mastitis.

Recent studies have revealed that mastitis is associated to a dysbiosis state in the mammary gland, which is characterized by an overrepresentation of staphylococci and a depletion of other bacterial groups, including lactobacilli and strict anaerobes^14–17^. The results of this study confirm a drastic change in the microbiological profile of milk samples from mastitis women. This change was mainly related to an increase in the bacterial load rather than to the type of bacterial species present in the milk samples, except for *S. aureus.* This species had higher prevalence in samples from mastitis-suffering woman (52%) than in healthy controls (8%) and its load was more than 2.5 times (log_10_ CFU/mL) higher in MW samples than in samples from healthy women. In addition, *S. aureus* was detected in all samples and at higher amount (about 1.7-fold increase in log_10_ CFU/mL of *S. aureus* counts) from AM cases compared to SAM cases. This role of *S. aureus* as the main etiological agent of AM is in agreement with previous studies^18,19^.

A different pattern was observed for *S. epidermidis* since, in contrast to *S. aureus*, this species was detected in almost all the milk samples from both MW and HW groups, but its prevalence and abundance was much higher in SAM than in AM cases. *S. epidermidis* is a commensal inhabitant of skin and mucosal surfaces in healthy individuals and its presence is a characteristic feature of milk and feces of healthy women and their breastfed infants, respectively^20^. Being part of the human microbiota in healthy individuals, *S. epidermidis* also acts as a common nosocomial pathogen in certain circumstances and it is frequently involved in human subacute mastitis^14,16,18,21^.

Streptococci are relevant members of the milk microbiota in healthy women^15,22,23^, but, when their levels increase, they can also cause subacute and subclinical mastitis^21^. In this study, *S. mitis/oralis* and *S. salivarius* were detected in > 20% of the milk samples, and at higher numbers in samples from mastitis suffering women, although globally no differences were found neither in prevalence or load between AM and SAM samples. A detailed analysis of the individual bacterial profile of mastitis samples showed that streptococci were frequently isolated together with staphylococci, but also they were the predominant bacterial species found in a relatively low percentage (20%) of samples from SAM cases.

The results show bacterial changes in milk related to mastitis and bacterial differences between AM and SAM cases. We performed a cultured-based analysis of the milk samples because all the bacterial species involved in human mastitis grow well under simple and economical culture conditions, a fact that is of paramount relevance for in practice diagnosis in Clinical Microbiology laboratories. In addition, previous metataxonomic and metagenomic approaches form our group and others^15,17^ have already shown the ample dominance of reads corresponding to the etiological agents (*S. aureus* in AM cases and *S. epidermidis* in the SAM ones) therefore, there is a high correlation between the main findings using culture-dependent or culture-independent methods. Culture of milk samples and identification of isolates should be considered as the appropriate method to disclose the causal agent(s) for the correct etiological diagnosis of mastitis. In addition, it informs appropriate treatment when taking into account recent descriptions of high antimicrobial resistance among mastitis pathogens^18,24^.

Human milk contains a wide array of soluble immune factors, which varies according to environmental and host factors^6,25^. In this work, notable differences were observed in the prevalence and the concentration of 18 soluble immune factors, reflecting a clear inflammatory response in samples from mastitis cases. IL2, a potent regulator of the growth and differentiation of lymphocytes T controlling the Th1/Th2 differentiation and the adaptive immune response against bacteria^26^, was detected only in samples from the mastitis group.

INFγ, which is the main macrophage-activating cytokine and has fundamental functions in innate immunity and adaptive cellular immunity against intracellular microorganisms^27^, was present, and at high concentration, in 96% of samples from mastitis samples. In contrast, no differences were observed in the prevalence and concentration of IL12 between samples from healthy and mastitis women. IL12 coordinates, together with INFγ, the link between pathogen recognition by innate immune cells and the induction of specific immunity^28^. Other proinflammatory cytokines (TNFα, IL1β, IL8, and MCP1 with different roles in the host's immune response to infection^29,30^) were detected in practically all samples from mastitis cases and at higher levels (eightfold, sevenfold, fourfold and twofold increase, respectively) than those from healthy women, in agreement with previous reports^31,32^. IL8 deserves special mention because is a potent chemoattractant recruiting immune cells to the site of inflammation, and it has been proposed as an effective indicator of mastitis both in human and ovine milk^31,33^. The fact that IL8 is present in practically all human milk samples analysed in this and other studies on human milk cytokines^6^ together with the substantial increase in its level (about fourfold in its median concentration) linked to mastitis supports its value as mastitis biomarker. However, in this study, IL8 concentration in milk did not have adequate discriminatory ability for mastitis in women as indicated the constructed ROC (Receiver Operating Characteristic) curve and the calculated AUC (Area Under the Curve; AUC [95% CI] = 0.747 [0.649–0.845]; Supplementary Fig. 5).

Human milk also contains several anti-inflammatory cytokines, such as IL4, IL10, IL13, and TGFβ_2_^3,34^. IL10 is one of the most potent anti-inflammatory cytokines, acting on the activated macrophages to terminate the inflammatory response and return the system to its resting state when the microbial infection has been eradicated. IL10 not only repress the production of pro-inflammatory cytokines (TNFα, IL1β, IL6) by activated macrophages, similarly to IL13, but it also regulates the expression of their receptors. In this study, these anti-inflammatory cytokines were less prevalent and their concentrations (mainly IL10 and IL13) were lower in samples from mastitis cases than in samples from healthy women in line with the observations of Tuaillon et al*.*^32^. However, the immunological profile of milk samples revealed remarkable differences, mainly related to the components of the adaptive immune system, depending on the etiological agent of mastitis, with IL10 and IL13 being detected in the samples from SAM cases but not in those of AM cases. Globally, the lower level of pro-inflammatory cytokines and the increased content of IL10 registered in milk from SAM cases has been claimed to explain the subacute nature and persistence of *S. epidermidis* infection^35^. *S. epidermidis* is a common commensal in humans from very early in life^20^, but it is also one of the main SAM etiological agents as it has been revealed in this and previous studies^21,24^. *S. epidermidis* biofilm-grown strains induce attenuation in phagocytic function and elicit production of anti-inflammatory rather than pro-inflammatory cytokines^36^. In contrast, *S. aureus,* which is also both a human commensal and a pathogen and was present at high levels (> 4 log_10_ CFU/mL) in all AM samples, displays a diversity of virulence factors that explain the acute course of its infection^37^.

It should be highlighted that mastitis did not modify the levels of TGFβ2, the most abundant cytokine in milk. TGFβ2 is secreted in a latent state that requires activation at the infant gastrointestinal tract, where it plays a protective role of the gut mucosa, and develops and maintains appropriate immune responses^25,38,39^. The stability of TGFβ2 levels in mastitis milk samples, would support, at least partially, that mastitis does not constitute a problem for the lactating infant. Moreover, breastfeeding is encouraged in this condition to maintain an adequate milk supply. SAM does not seem to affect infant growth and development at the long term^40^. This observation is important because breastfeeding provides many non-nutritional health benefits to infants including passive immunological protection during this period of host defence vulnerability, contributes to the active maturation and shaping of the immature infant’s immune system and mucosal barrier, and fosters the correct infant psychological development through emotional bonding.

In this study, we also explored potential relationship between HMOs, the main etiological agents of mastitis and the immunological profile of milk samples. The Secretor status has a genetic basis related to the presence of the gene encoding the α1-2-fucosyltransferase 2 (FUT2) enzyme which is a dominant trait. FUT2 transfers fucose to the 2 position of β-galactose and it is required for the synthesis of 2’-fucosyllactose (2’FL), the most abundant HMO in Secretor women^4^. FUT2, as well as other glucosyltransferases responsible for the synthesis of HMOs in the mammary gland, are also involved in the glycosylation of cell membranes in epithelial cells. Therefore, the Secretor status may influence the adhesion of pathogens and toxins because some microorganisms attach to specific glycosylated motifs on the cell membrane structures; in the particular case of human milk and other body secretions, HMOs may act as soluble receptors for different microorganisms and toxins blocking their attachment to the cellular surface^41^. In fact, modifications in cell glycosylation pattern have the potential to change the susceptibility to develop numerous infections and other pathologies such as cancer and metabolic diseases^42^. Individuals with non-Secretor status, carrying null mutations in the *FUT2* gene have been associated with higher susceptibility to some pathogens such as *Streptococcus pneumoniae*, *Streptococcus pyogenes*, *Haemophilus influenza*, *Helicobacter pylori*, *Neisseria meningitis*, and *Candida albicans*, among many others^43^. Although data is scarce in relation to staphylococci, non-Secretor status was associated to an increased risk (6.5 times) of carrying *S. aureus* in the throat than Secretors^44^. In contrast, Secretors are at higher risk of infections associated to other pathogens that require glycosylated receptors related to FUT2 activity, mainly *Norovirus*, *Rotavirus, Coronavirus,* HIV…) and susceptibility to some types of cancers and chronic diseases^43,45^. In this study, a high percentage of the participants (81%) were Secretors, in agreement with other studies^46^, but there was not association between the Secretor status of the woman and a higher risk of developing mastitis or the type of mastitis (SAM or AM). In addition, high IL7 (which is involved in lymphocyte development and maintenance, and can cross the intestinal barrier) and TGFβ_2_ milk levels in non-Secretor women, may influence the differential susceptibility of infants from Secretor and non-Secretor mothers to infectious and autoimmune and chronic inflammatory diseases^43,45,47^.

Recent studies have explored the association of maternal Secretor status on the HMO and microbiological profiles of human milk using culture-independent methodology^48^. In our study, differences in the milk bacterial and HMO content were noted among healthy women depending on the Secretor status. First, the concentration of total bacterial counts and, more specifically that of *S. epidermidis,* which is the most frequent and abundant bacterial species in human milk, were higher in samples from non-Secretor than in those from Secretor healthy women. In fact, the total bacterial and *S. epidermidis* load had a strong negative correlation with both small HMOs (fucosylated and sialylated lactose at different linkage positions) and the total amount of fucose linked to HMOs. These results indicate a potential role of the human milk microbiota in the HMO content or vice versa. However, this difference was only registered in healthy women but not in mastitis women, as this condition lead to a substantial change of most bacterial species present in milk.

Apart from nourishing health-promoting bacteria in the gut of breastfed infants and reducing pathogen attachment and infectivity by antiadhesive features^49^, emerging research suggest that HMOs may affect infant immunity by interacting with receptors located in intestinal immune cells^9^. In our study, we could find a profile of HMOs clearly associated with the course of mastitis. The concentration of sialylated, type 2 and α2, 6-sialylated HMOs, and more specifically LNFP I, LNFP III, 6’SL, LSTc, DSLNT and LNH, was higher among samples from Secretor women that were suffering from mastitis than in healthy women, while LNFP II, DFLNH and LSTb were more abundant among Secretor healthy women. Although the specific interactions between HMOs and human milk microbiota are largely unknown, some studies have observed that certain HMOs by serving as metabolic substrates for bifidobacteria contribute to shape the composition of the infant gut microbiota^50–53^. In contrast, there are few studies addressing the prebiotic potential of HMOs for other species usually present in human milk. Interestingly, a study showed that some HMOs stimulated the growth of *S. aureus* and *S. epidermidis* strains isolated from human milk although they did not metabolize them^54^. In addition, the presence of *Staphylococcus* and *Streptococcus* in the faeces of infants increased in relation to high consumption of fucosylated HMOs (DFLac, LNFP-II and LNFP-III)^55^. In our study we found different relationships between HMOs, immunological compounds and human milk microbiota as a function of the women’s health status, which should be explored further. *S. epidermidis* was negatively correlated with the concentrations of small and fucosylated HMOs, and in particular with those of 2’FL, DFLac and DFLNT, in healthy women but not in women with mastitis. In contrast, in this group, only weak positive correlations were observed between *S. epidermidis* and the most abundant neutral HMO (LNT) and between *S. aureus* and two sialylated HMOs, 6’SL and FDSLNH. A strong positive correlation between *S. aureus* and FDSLNH has been previously reported^56^. Through changes in the composition of the microbiota, HMOs indirectly interfere with the immune system, but some recent in vitro and in vivo studies suggest that they can also directly influence the production of immunological compounds^57–59^.

Some biological functions of HMOs are specific to their structure^49^, but the great structural diversity, variable concentration and lack of knowledge of HMOs biosynthetic pathways have limited the accessibility to an adequate amount of purified individual HMOs for structure–function linkage studies. In addition, the scarce available studies of purified HMOs have focussed on their impact on infant health^60^, and the immunological effect of HMOs in the mammary gland is unknown. The literature suggests that immunomodulatory effects of HMOs are usually linked to anti-inflammatory function^61^. Our study showed that the mastitis immunological profile in the mammary gland may be related to specific HMOs, as the levels of the branched hexasaccharide LNH, its disialylated derivative DSLNH, 3’SL, and DFLac were positively associated to some pro-inflammatory cytokines (IL2, IL6, IL17 and IFNγ). But different relationships were noted in healthy women: of the three most abundant fucosylated HMOs, one (2’FL) was negatively associated to IL7 and TGFβ2, while the other two (3FL and LNFP II) showed a positive association. However, more studies are needed to clarify the role that HMOs may have in the susceptibility or resistance to develop mastitis and in the immunological course of this condition in breastfeeding women.

Overall, the findings of this study reinforce the hypothesis that microorganisms, immune compounds and HMOs interact with each other in the mammary gland. These complex interactions take a drastic turn when a woman experiences mastitis and the changes seem to depend on the etiological agent and the course of the infection. Understanding these interactions in depth can provide information about a woman's predisposition to suffering mastitis and the development and the course of this condition. In addition, it may provide a new piece of information to understand the influence of human milk in the infant health outcomes.

## Materials and methods

### Sampling

A total of 110 breastfeeding women were enrolled in this study, including 69 showing clinical symptoms of mastitis (MW group) and 41 healthy women (HW group). Mastitis cases were classified as either subacute (SAM) or acute (AM) mastitis, according to the criteria of Fernández et al.^14^. When women reported bilateral infection (n = 21), the sample from the most affected breast was collected for analysis. To be eligible for inclusion as members of the HW group, women had to report that they had an uncomplicated pregnancy, their child was born at term and they were breastfeeding a healthy infant without any complications, including the absence of symptoms related to breast infection or breast pain from birth to recruitment. HW provided a single sample which was collected from the breast that the mother chose. In both cases (HW and MW groups), use of antibiotics or probiotics in the previous 14 days were excluded. All volunteers gave written informed consent to the protocol (reference 10/017E), which was approved by Ethical Committee of Hospital Clínico San Carlos (Madrid, Spain). The study was carried out in accordance with the Declaration of Helsinki.

Medical staff or the mother, under the supervision of medical staff, wearing sterile gloves, collected the milk sample aseptically in a sterile polypropylene tube by manual expression. Previously, nipples and mammary areola had been cleaned with soap and water. Samples were immediately frozen (− 20 °C) until analysis.

### Milk cultures and identification of the isolates

Milk samples were plated onto Columbia Nadilixic Acid (CNA; BioMérieux, Marcy l’Etoile, France; for isolation of staphylococci, streptococci, enterococci and taxonomically-related Gram-positive bacteria), MacConkey (MCK; BioMérieux: for isolation of enterobacteria), Wilkins Chalgren (WC; Oxoid, Basingstoke, UK for general isolation of anaerobic bacteria) and de Man, Rogosa and Sharpe (MRS; Oxoid) supplemented with 0.25% L-cysteine (Sigma-Aldrich, St. Louis, USA) (MRSCys; for isolation of lactic acid bacteria and bifidobacteria) agar plates to identify and quantify the viable bacteria present in the samples. They were also plated on Sabouraud Dextrose Chloramphenicol (SDC, BioMérieux) for isolation of yeasts. CNA, MCK and SDC plates were incubated in aerobic conditions at 37 °C for 48 h while WC and MRSCys were incubated anaerobically (85% nitrogen, 10% hydrogen, 5% carbon dioxide) at 37 °C for 48 h in an anaerobic workstation (DW Scientific Shipley, UK). The isolates were identified by Matrix-Assisted Laser Desorption Ionization-Time of Flight (MALDI-TOF) mass spectrometry as described previously^18^.

### HMO analysis

HMO analysis was conducted at the University of California San Diego using established methods^5^. Briefly, human milk (20 μL) was spiked with raffinose (a non-HMO carbohydrate) as an internal standard at the beginning of sample preparation to correct for sample losses during sample processing and allow for absolute oligosaccharide quantification. Oligosaccharides were extracted by high-throughput solid phase extraction over C18 (Hypercarb-96, 25 mg bed weight, Thermo Scientific) and Carbograph microcolumns (Hypersep-96 C18, 25 mg bed weight, Thermo Scientific) using a controlled vacuum manifold. Use of high-throughput microcolumns was validated in multiple different ways: (1) establishing parallelism in serial dilutions, (2) spiking milk with individual HMO standards to determine recovery, and (3) comparison with direct in-sample derivatization as used by others^62^. Oligosaccharides were fluorescently labelled with 2-aminobenzamide (2AB, Sigma) in a 96-well thermocycler at 65 °C for exactly 2 h. The reaction was stopped abruptly by reducing the thermocycler temperature to 4 °C. The amount of 2AB was titrated to be in excess to account for the high and variable amount of lactose and other glycans in milk samples. Unreacted 2AB was removed by high-throughput solid phase extraction over silica microcolumns (Hypersep silica, 25 mg bed weight, Thermo Scientific). Labeled oligosaccharides were analyzed by HPLC (Dionex Ultimate 3000, Dionex, now Thermo Scientific) on an amide-80 column (15 cm length, 2 mm inner diameter, 3 μm particle size; Tosoh Bioscience) with a 50-mmol/L ammonium formate–acetonitrile buffer system. Separation was performed at 25 °C and monitored with a fluorescence detector at 360 nm excitation and 425 nm emission. Peak annotation was based on standard retention times of commercially available HMO standards (Sigma, Dextra, Elicityl) and a synthetic HMO library^63^ and offline mass spectrometric analysis on a Thermo LCQ Duo Ion trap mass spectrometer equipped with a Nano-ESI-source. Absolute concentrations were calculated based on HMO standard response curves for each of the annotated HMO. (Oligosaccharide detection limit: ~ 20 pmol, dynamic range between 20 and 5000 pmol; milk samples were diluted accordingly). The total concentration of HMOs was calculated as the sum of the annotated oligosaccharides. The proportion of each HMO making up the total HMO concentration was also calculated. HMO-bound fucose was calculated on a molar basis. One mole HMO with one fucose residue counted as one mole HMO-bound fucose while one mole HMO with two or more fucose residues counted as two or more moles of HMO-bound fucose. The same was calculated for HMO-bound sialic acid. Maternal Secretor status was determined by the high abundance (Secretor) or near absence (Non-Secretor) of the HMO 2’-fucosyllactose in the respective milk samples with a cutoff of 100 nmol 2’FL/mL.

### Immunological analysis

The concentrations of 18 immune factors, including innate immune factors (IL1β, IL6, IL12, IFNγ, TNFα), acquired immunity factors (IL2, IL4, IL10, IL13, IL17), chemokines (IL8, MCP1, MIP1β), and growth factors (IL5, IL7, GCSF, GMCSF, TGFβ2) were determined by magnetic bead-based multiplex immunoassays, using a Bioplex 200 instrument (Bio-Rad, Hercules, CA, USA) and the Bio-Plex Pro Human Cytokine and Bio-Plex Pro TGFβ assays (Bio-Rad), according to manufacturer’s instructions.

Prior to their analysis, milk samples (1 mL) were centrifuged (13,000 rpm, 15 min, 4 °C). After defatting, the supernatant was aliquoted in different tubes for subsequent immunological analysis. A fresh aliquot was used for each assay, avoiding freeze–thaw cycles. Every assay was run in duplicate according to manufacturer’s instructions, and standard curves were performed for each analyte on every assay. Concentration of all the immune compounds were expressed as ng/L with the exception of those of TGFβ_2_, which were expressed as µg/L.

### Statistical analyses

Distribution of the data was evaluated using Shapiro–Wilk normality test. Data with normal distribution was expressed as mean and 95% confidence interval (CI), and data with non-normal distribution was expressed as median and interquartile range (IQR). Microbiological data recorded as colony-forming units (CFU) per mL of milk, HMO quantities and immunological compounds concentrations were log_10_ transformed before analyses.

Chi-square test was performed aiming to check if the Secretor status was related to mastitis. The effect of the group (MW/HW or Secretor/non-Secretor) on the concentrations of microorganisms, immunological compounds and oligosaccharides was tested using one-way ANOVA or Wilcoxon sum rank tests (depending on the actual data distribution). In the cases of microorganisms and immunological compounds, differences in the frequency of detection among groups were tested using Chi-squared or Fisher’s exact tests. Wilcoxon sum rank tests was used to identify differences in concentrations of HMOs and immunological compounds between AM and SAM cases. False Discovery Rate was used to correct *p*-values in each analysis.

Heatmaps of the different datasets (microbiological counts and concentrations of HMO and immune factors) were performed to visualize the data profiles and the existence of potential grouping according to the Secretor/non-Secretor and/or mastitis/healthy status.

Receiver-operator characteristic (ROC) analysis was performed with IL8 concentration against mastitis. The area under ROC curve (AUC), which was estimated to assess the predictive power of IL8 concentration for mastitis, was calculated using IBM SPSS Statistics 25 software (SPSS Inc., Chicago, IL, USA).

Significance for all statistical tests was declared at *p* ≤ 0.05. All statistical analyses, except ROC analysis and AUC calculation, have been performed with the software R statistic, version 4.0.2 (R-project, http://www.r-project.org).

## Supplementary Information

Supplementary Information.
