# Supplementary material for: Interactions between human milk oligosaccharides, microbiota and immune factors in milk of women with and without mastitis
Source: Sci Rep. 2022 Jan 25;12:1367. doi: 10.1038/s41598-022-05250-7 (PMC8789856; doi:10.1038/s41598-022-05250-7)
Supplement: Supplementary file 1 — Supplementary Information. [file 41598_2022_5250_MOESM1_ESM.docx]

**Interactions between human milk oligosaccharides, microbiota and immune factors in milk of women with and without mastitis**

Irma Castro^1^, Cristina García-Carral^1,2^, Annalee [Furst](mailto:aloeffler@health.ucsd.edu)^3^, Sadaf Khwajazada^3^, Janneiry García^3^, Rebeca Arroyo^1^, Lorena Ruiz^4^, Juan M. Rodríguez^1^, Lars Bode^3,*^, Leónides Fernández^5,*^

^1^Departament of Nutrition and Food Science, Complutense University of Madrid, Madrid, Spain

^2^Probisearch S.L., Tres Cantos, Spain

^3^Department of Pediatrics and Larsson-Rosenquist Foundation Mother-Milk-Infant Center of Research Excellence, University of California San Diego, La Jolla, California, USA

^4^IPLA-CSIC, Department of Microbiology and Biochemistry of Dairy Products, Institute of Dairy Products of Asturias, Villaviciosa, Spain

^5^Department of Galenic Pharmacy and Food Technology, Complutense University of Madrid, Madrid, Spain

Correspondence and requests for materials should be addressed to L.B. (email: lbode@helath.ucsd.edu) or L.F. (email: leonides@vet.ucm.es).

**Supplementary Table 1.** Concentration (nmol/mL) of grouped HMOs in milk samples according to the Secretor status and breast health status (HW, healthy women; MW, mastitis cases) in women.

| **HMO** | **NON-SECRETOR** | | | **SECRETOR** | | | |
| --- | --- | --- | --- | --- | --- | --- | --- |
|  | **HW (n = 10)** | **MW (n = 11)** | ***p-*value^1^** | | **HW (n = 31)** | **MW (n = 62)** | ***p-*value^1^** |
|  | **Median (IQR) or** | **Median (IQR) or** |  |  | **Median (IQR) or** | **Median (IQR) or** |  |
|  | **mean [95% CI]** | **mean [95% CI]** |  |  | **mean [95% CI]** | **mean [95% CI]** |  |
| HMO-bound sialic acid^2^ | 1445.02 [454.63] | 1814.97 [530.32] | 0.261 | | 1494.50 (1092.00-1821.95) | 2209.05 (1508.55-2927.97) | *0.000 |
| HMO-bound fucose acid^3^ | 11953.85 (9840.57-13970.92) | 11236.40 (9084.85-11751.40) | 0.387 | | 15179.80[898.38] | 14959.79 [577.08] | 0.912 |
| Small HMOs^4^ | 6582.75 [1762.07] | 6101.08 [1471.87] | 0.774 | | 9782.40 [578.31] | 9971.63 [541.59] | 0.665 |
| Type 1 HMOs^5^ | 5199.25 [1348.77] | 4299.32 [1296.04] | 0.212 | | 3864.42 [467.61] | 3703.35 [373.18] | 0.851 |
| Type 2 HMOs^6^ | 182.95 (154.32-283.57) | 254.40 (195.70-401.90) | 0.314 | | 398.90 (335.65-528.30) | 653.65 (416.85-934.15) | *0.000 |
| α-1,2^7^ | 223.49 [61.03] | 242.16 [69.77] | 0.693 | | 7178.72 [874.60] | 8072.34 [785.43] | 0.655 |
| α-1,3^8^ | 5725.79 [1666.11] | 4978.54 [1348.48] | 0.636 | | 2218.20 (1530.40-3201.40) | 1726.30 (1288.82-2786.97) | 0.313 |
| α-2,6^9^ | 468.65 (279.52-671.65) | 905.50 (716.85-1160.05) | 0.116 | | 416.00 (280.30-638.70) | 1010.00 (585.32-1269.26) | *0.000 |
| Evenness | 0.21 [0.04] | 0.21 [0.04] | 0.324 | | 0.25 (0.20-0.30) | 0.23 (0.19-0.30) | 0.750 |
| Diversity | 4.15 [0.96] | 4.03 [0.89] | 0.324 | | 4.75 (3.75-5.70) | 4.40 (3.55-5.72) | 0.750 |
| Total HMOs | 15183.95 (12131.33-15836.43) | 13948.00 (10163.20-12470.15) | 0.114 | | 16039.70 (14288.35-18290.85) | 17283.75 (14594.78-18473.79) | 0.534 |

^1^Wilcoxon rank sum test or ANOVA tests (depending on the distribution of the data) were used to determine differences in HMOs concentration between samples from mastitis-suffering or healthy women from Secretor or non-Secretor status. FDR-adjusted *p*-values.

*Statistically significant difference, *p* < 0.05.

^2^Calculated as the sum of all sialic acid moieties bound to each HMO.

^3^Calculated as the sum of all fucose moieties bound to each HMO.

^4^Calculated as 2'FL + 3FL + 3'SL + 6'SL.

^5^Calculated as LNT + LNFP I + LNFP II + LSTb + DSLNT.

^6^Calculated as LNnT + LNFP III + LSTc.

^7^Calculated as LNFP I + 2’FL

^8^Calculated as LNFP III + 3FL.

^9^Calculated as LSTc + 6'SL.

**Supplementary Table 2.** Microbiological counts (expressed as log_10_ CFU/mL) using culture-dependent analysis of milk samples from healthy women (HW, n = 37) and women with mastitis (MW; n = 69) according to their Secretor status.

|  | **HW** | | | |  |  | | **MW** | | | | | |  | |  | |
| --- | --- | --- | --- | --- | --- | --- | --- | --- | --- | --- | --- | --- | --- | --- | --- | --- | --- |
| **Microorganism** | **Non-Secretor (n = 9)** | | **Secretor (n = 28)** | |  |  | **Non-Secretor (n = 11)** | | | **Secretor (n = 58)** | | |  | |  | |  |
|  | **n (%)^1^** | **Median (IQR)^2^** | **n (%)** | **Median (IQR)** | ***p*-value^3^** | ***p*-value^4^** | | **n (%)** | **Median (IQR)** | | **n (%)** | **Median (IQR)** | | ***p*-value^3^** | | ***p*-value^4^** | |
| **Firmicutes** |  |  |  |  |  |  | |  |  | |  |  | |  | |  | |
| *Staphylococcus epidermidis* | 9 (100) | 2.86 (2.58-3.38) | 26 (93) | 2.10 (1.60-2.71) | 1.000 | 0.063 | | 8 (73) | 5.05 (4.79-5.26) | | 51 (88) | 5.00 (4.52-5.33) | | 0.975 | | 1.000 | |
| *Staphylococcus aureus* | 2 (22) | 1.65 (1.44-1.80) | 1 (4) | 1.30 | 0.890 | 1.000 | | 8 (73) | 4.68 (3.38-5.08) | | 31 (53) | 4.88 (4.10-5.30) | | 0.975 | | 1.000 | |
| Other staphylococci^5^ | 1 (11) | 2.30 | 4 (14) | 2.60 (1.89-2.92) | 1.000 | 1.000 | | 3 (27) | 2.65 (2.57-3.60) | | 5 (9) | 4.65 (3.70-4.70) | | 0.545 | | 0.786 | |
| *Streptococcus mitis/oralis* | 4 (44) | 1.70 (1.40-2.09) | 5 (18) | 2.46 (1.00-2.90) | 0.890 | 1.000 | | 4 (36) | 4.65 (4.14-4.94) | | 15 (26) | 3.70 (3.33-5.09) | | 0.975 | | 1.000 | |
| *Streptococcus salivarius* | 1 (11) | 2.60 | 3 (11) | 1.84 (1.60-1.93) | 1.000 | 1.000 | | 4 (36) | 4.51 (4.06-4.70) | | 14 (26) | 4.14 (3.70-4.62) | | 0.975 | | 1.000 | |
| Other streptococci^6^ | 4 (44) | 1.81 (1.63-2.10) | 9 (32) | 1.95 (1.30-2.08) | 1.000 | 1.000 | | 1 (9) | 4.30 | | 11 (19) | 4.00 (3.70-4.70) | | 0.975 | | 1.000 | |
| *Enterococcus* spp. | 1 (11) | 3.83 | 1 (4) | 1.00 | 0.990 | 1.000 | | 2 (18) | 4.73 (4.48-4.88) | | 1 (2) | 4.18 | | 0.480 | | 1.000 | |
| Other Firmicutes^7^ | 3 (33) | 2.50 (2.41-3.06) | 4 (14) | 2.04 (1.44-3.27) | 0.990 | 1.000 | | 3 (27) | 4.40 (4.35-4.51) | | 3 (5) | 3.78 (3.63-3.90) | | 0.480 | | 0.786 | |
| **Proteobacteria**^8^ | 3 (33) | 3.60 (3.31-3.73) | 0 (0) | - | 1.000 | - | | 0 (0) | - | | 5 (9) | 2.60 (2.54-5.10) | | 0.975 | | - | |
| **Actinobacteria** |  |  |  |  |  |  | |  |  | |  |  | |  | |  | |
| *Rothia.mucilaginosa* | 1 (11) | 1.78 | 3 (11) | 1.78 (1.54-1.84) | 1.000 | 1.000 | | 2 (18) | 3.72 (3.46-3.88) | | 7 (12) | 3.78 (3.46-4.97) | | 0.975 | | 1.000 | |
| *Corynebacterium* spp. | 3 (33) | 1.00 (1.00-1.18) | 2 (7) | 1.18 (1.10-1.24) | 0.890 | 1.000 | | 1 (9) | 3.40 | | 8 (14) | 3.70 (3.62-4.19) | | 1.000 | | 1.000 | |
| Other Actinobacteria^9^ | 2 (22) | 2.10 (1.86-2.25) | 7 (25) | 2.60 (1.30-3.10) | 1.000 | 1.000 | | 0 (0) | - | | 0 (0) | - | | 1.000 | | - | |
| **Yeast** | 1 (11) | 2.30 | 1 (4) | 1.00 | 0.990 | 1.000 | | 0 (0) | - | | 4 (7) | 3.14 (2.85-4.15) | | 0.975 | | - | |
| **Not identified** | 6 (67) | 2.86 (2.38-3.15) | 14 (50) | 2.35 (2.01-2.59) | 0.990 | 0.933 | | 0 (0) | - | | 0 (0) | - | | 1.000 | | - | |
| **Total CFU** | 9 (100) | 3.10 (2.96-3.69) | 28 (100) | 2.38 (1.67-3.04) | 1.000 | 0.063 | | 11 (100) | 5.34 (5.03-5.47) | | 58 (100) | 5.31 (5.04-5.54) | | 1.000 | | 1.000 | |

^1^n (%): number (percentage) of samples in which the microorganism was detected (relative frequency of detection).

^2^All data expressed as median (IQR) log_10_ CFU/mL (only samples where bacterial growth was detected).

^3^Fisher tests were used to determine a possible association between the Secretor or non-Secretor status and woman health (HW and MW groups) in those women in which the microorganism or group was isolated. FDR-adjusted *p*-values.*Statistically significant difference, *p* < 0.05.

^4^Wilcoxon rank sum tests were used to determine if there were differences in microbiological counts according to Secretor status in HW and MW groups. FDR-adjusted *p*-values.

^5^Other staphylococcal species that were identified include *S. hominis*, *S. lugdunensis,* *S. pasteuri* and *S. warneri*.

^6^Other streptococcal species that were identified include *S. anginosus*, *S. gordonii,* *S. parasanguinis, S. pneumoniae* and *S. vestibularis.*

^7^Other Firmicutes includes *Bacillus*, *Lactococcus*, former *Lactobacillus* and *Weisella.*

^8^Proteobacteria includes *Brevundimonas*, *Enterobacteriaceae, Moraxella, Rhizobium* and *Stenotrophomonas*.

^9^Other Actinobacteria includes *Actinomyces, Bifidobacterium*, *Cutibacterium*, *Kocuria* and *Propionibacterium.*

IQR: interquartile range; CFU: colony-forming units.

**Supplementary Table 3.** Frequency and concentration of immune factors in milk samples according to the breast health status (HW, healthy women; MW, mastitis cases) and secretory status.

|  | **HW** | | | | | | | | | **MW** | | | | | | | | |  |
| --- | --- | --- | --- | --- | --- | --- | --- | --- | --- | --- | --- | --- | --- | --- | --- | --- | --- | --- | --- |
|  | **Non-Secretor (n=10)** | | **Secretor (n=31)** | | |  | |  | | **Non-Secretor (n=8)** | | | **Secretor (n=46)** | | |  | |  |  |
|  | **n (%)^1^** | **Median (IQR)^2^** | **n (%)** | **Median (IQR)** | ***p-*value^3^** | | ***p-*value^4^** | | **n (%)** | | **Median (IQR)** | **n (%)** | | **Median (IQR)** | ***p-*value^3^** | | ***p-*value^4^** | | |
| **Innate immunity** |  |  |  |  |  | |  | |  | |  |  | |  |  | |  | | |
| IL1β (ng/L) | 9 (90) | 0.40 (0.30-1.50) | 14 (45) | 1.35 (0.62-6.72) | 0.225 | | 0.479 | | 7 (87) | | 3.21 (1.37-16.10) | 44 (96) | | 8.24 (1.17-23.67) | 1.000 | | 1.000 | | |
| IL6 (ng/L) | 7 (70) | 12.70 (8.35-18.05) | 10 (32) | 13.15 (7.77-18.05) | 0.373 | | 1.000 | | 6 (75) | | 13.12 (4.51-31.10) | 36 (78) | | 10.26 (2.10-37.43) | 1.000 | | 1.000 | | |
| IL12(p70) (ng/L) | 1 (10) | 0.10 | 5 (16) | 0.90 (0.20-0.90) | 1.000 | | 0.479 | | 2 (25) | | 4.62 (3.56-5.69) | 6 (13) | | 1.42 (0.76-3.52) | 1.000 | | 1.000 | | |
| IFNγ (ng/L) | 0 (0) | - | 1 (3) | 4.70 | 1.000 | | - | | 8 (100) | | 79.44 (21.17-295.30) | 44 (96) | | 54.43 (11.22-259.90) | 1.000 | | 1.000 | | |
| TNFα (ng/L) | 4 (40) | 3.45 (2.52-4.75) | 14 (45) | 3.10 (2.02-4.52) | 1.000 | | 0.886 | | 8 (100) | | 35.24 (16.53-95.36) | 46 (100) | | 29.02 (12.34-67.72) | 1.000 | | 1.000 | | |
| **Acquired immunity** |  |  |  |  |  | |  | |  | |  |  | |  |  | |  | | |
| IL2 (ng/L) | 0 (0) | - | 0 (0) | - | 1.000 | | - | | 4 (50) | | 15.46 (11.63-19.40) | 34 (74) | | 5.95 (0.98-17.51) | 1.000 | | 1.000 | | |
| IL4 (ng/L) | 1 (10) | 0.70 | 0 (0) | - | 0.878 | | - | | 3 (37) | | 1.01 (0.85-1.31) | 25 (54) | | 0.55 (0.26-1.32) | 1.000 | | 1.000 | | |
| IL10 (ng/L) | 8 (80) | 3.75 (2.70-4.15) | 24 (77) | 3.15 (1.60-4.40) | 1.000 | | 0.886 | | 0 (0) | | - | 6 (13) | | 3.51 (3.29-4.40) | 1.000 | | - | | |
| IL13 (ng/L) | 8 (80) | 4.20 (3.20-7.60) | 25 (81) | 2.40 (1.30-2.90) | 1.000 | | 0.266 | | 1 (12) | | 0.22 | 9 (19) | | 0.76 (0.76-1.24) | 1.000 | | 1.000 | | |
| IL17 (ng/L) | 0 (0) | - | 2 (6) | 4.30 (3.20-5.40) | 1.000 | | - | | 5 (62) | | 21.42 (7.86-31.70) | 26 (56) | | 17.67 (9.89-52.57) | 1.000 | | 1.000 | | |
| **Chemokines** |  |  |  |  |  | |  | |  | |  |  | |  |  | |  | | |
| IL8 (ng/L) | 10 (100) | 128.55 (95.47-184.25) | 31 (100) | 43.60 (22.40-152.85) | 1.000 | | 0.343 | | 8 (100) | | 199.70 (172.50-1321.00) | 46 (100) | | 275.70 (98.84-1818.00) | 1.000 | | 1.000 | | |
| MCP1 (ng/L) | 10 (100) | 181.85 (136.77-313.50) | 22 (71) | 144.15 (51.67-265.80) | 0.373 | | 0.470 | | 8 (100) | | 511.00 (315.90-1646.00) | 45 (98) | | 375.70 (70.77-985.40) | 1.000 | | 1.000 | | |
| MIP1β (ng/L) | 10 (100) | 46.25 (36.37-103.47) | 29 (93) | 28.50 (14.60-55.00) | 1.000 | | 0.327 | | 8 (100) | | 31.68 (12.17-156.30) | 46 (100) | | 25.35 (9.75-161.30) | 1.000 | | 1.000 | | |
| **Hematopoyetic factors** | |  |  |  |  | |  | |  | |  |  | |  |  | |  | | |
| IL5 (ng/L) | 1 (10) | 2.80 | 2 (6) | 1.70 (1.25-2.15) | 1.000 | | 0.887 | | 3 (37) | | 21.32 (16.18-38.56) | 12 (26) | | 37.33 (19.74-67.32) | 1.000 | | 1.000 | | |
| IL7 (ng/L) | 10 (100) | 57.70 (42.40-87.57) | 28 (90) | 32.85 (25.45-51.30) | 1.000 | | 0.226 | | 4 (50) | | 21.71 (13.62-57.67) | 16 (35) | | 22.42 (11.47-62.17) | 1.000 | | 1.000 | | |
| GCSF (ng/L) | 10 (100) | 40.50 (18.42-71.77) | 19 (61) | 16.70 (4.90-38.30) | 0.225 | | 0.410 | | 7 (87) | | 176.50 (67.13-683.80) | 44 (96) | | 213.90 (47.61-692.00) | 1.000 | | 1.000 | | |
| GMCSF (ng/L) | 1 (10) | 12.90 | 2 (6) | 12.10 (6.25-17.95) | 1.000 | | 1.000 | | 4 (50) | | 1.54 (1.25-1.75) | 15 (33) | | 4.88 (2.03-5.23) | 1.000 | | 1.000 | | |
| TGFβ2 (µg/L) | 10 (100) | 2.55 (1.65-4.45) | 31 (100) | 1.80 (1.10-3.25) | 1.000 | | 0.410 | | 8 (100) | | 4.33 (1.20-6.53) | 46 (100) | | 1.31 (0.38-4.86) | 1.000 | | 1.000 | | |

^1^n (%): number (percentage) of samples in which the immunological compound was detected (relative frequency of detection)^.^

^2^Data expressed as median and interquartile range (IQR).

^3^Fisher tests were used to determine a possible association between the Secretor status and the immunological compound detected. FDR-adjusted *p*-values.

^4^Wilcoxon rank sum tests were used to determine differences in the concentration detected of each immunological compound according to Secretor status. FDR-adjusted *p*-values.

GCSF, granulocyte colony-stimulating factor; GMCSF, granulocyte-macrophage colony-stimulating factor; INFγ, interferon-γ; IL, interleukin; MCP1, macrophage-monocyte chemoattractant protein-1; MIP1β, macrophage inflammatoryprotein-1β; TGFβ2, transforming growth factor-β2; TNFα, tumor necrosis factor-α.

**Supplementary Table 4.** Microbiological counts (expressed as log_10_ CFU/mL) determined using culture-dependent analysis of milk samples from acute mastitis (AM; n = 29) and subacute mastitis (SAM; n = 40) cases.

| **Microorganism** | **AM (n = 29)** | | **SAM (n = 40)** | | |  | |  | |  |
| --- | --- | --- | --- | --- | --- | --- | --- | --- | --- | --- |
|  | **n (%)** | **Median (IQR)** | | **n (%)** | **Median (IQR)** | | ***p-*value^3^** | | ***p-*value^4^** | |
| **Firmicutes** |  |  | |  |  | |  | |  | |
| *Staphylococcus epidermidis* | 21 (72) | 4.70 (4.00-5.00) | | 38 (95) | 5.14 (4.87-5.40) | | 0.091 | | 0.156 | |
| *Staphylococcus aureus* | 29 (100) | 5.18 (4.78-5.30) | | 10 (25) | 3.06 (2.80-3.42) | | **0.000* | | *0.000 | |
| Other staphylococci | 4 (14) | 2.93 (2.61-4.45) | | 4 (10) | 4.42 (3.84-4.66) | | 0.974 | | 0.743 | |
| *Streptococcus mitis/oralis* | 10 (34) | 4.63 (3.47-5.05) | | 9 (22) | 4.18 (3.48-4.98) | | *0.772* | | 0.945 | |
| *Streptococcus salivarius* | 6 (21) | 4.18 (4.12-4.61) | | 12 (30) | 4.00 (3.57-4.70) | | *0.772* | | 0.743 | |
| Other streptococci | 6 (21) | 4.18 (3.80-4.63) | | 6 (15) | 4.38 (3.75-4.68) | | 0.974 | | 0.944 | |
| *Enterococcus* spp. | 1 (3) | 3.81 | | 2 (5) | 4.76 (4.56-4.90) | | 0.722 | | 0.910 | |
| Other Firmicutes | 3 (10) | 4.40 (4.14-4.51) | | 3 (7) | 4.00 (3.90-4.18) | | 1.000 | | 0.910 | |
| **Proteobacteria** | 1 (3) | 1.70 | | 4 (10) | 4.80 (2.59-5.10) | | 0.722 | | 0.743 | |
| *Rothia mucilaginosa* | 2 (7) | 5.37 (5.31-5.43) | | 7 (17) | 3.48 (3.21-3.90) | | 0.722 | | 0.195 | |
| *Corynebacterium* spp. | 4 (14) | 4.44 (3.60-5.00) | | 6 (15) | 3.70 (3.49-4.10) | | 1.000 | | 0.910 | |
| **Yeast** | 1 (3) | 2.87 | | 3 (7) | 3.30 (3.10-4.41) | | 0.974 | | 1.000 | |
| **Total CFU** | 29 (100) | 5.35 (5.21-5.54) | | 40 (100) | 5.24 (5.00-5.43) | | 1.000 | | 0.195 | |

^1^ n (%): number (percentage) of samples in which the microorganism was detected (relative frequency of detection).

^2^ All data expressed as median (IQR) log10 CFU/mL (only samples where bacterial growth was detected).

^3^ Fisher tests or χ2 tests (in italics) were used to determine a possible association between the type of mastitis and individual microorganisms or group of microorganisms isolated from milk samples. FDR-adjusted *p*-values. *Statistically significant difference, *p* < 0.05.

^4^ Wilcoxon rank sum tests were used to determine if there were differences in microbiological counts according to Secretor status in AM and SAM cases. FDR-adjusted *p*-values. *Statistically significant difference, *p* < 0.05.

^5^ Other staphylococcal species that were identified include *S. hominis*, *S. lugdunensis*, *S. pasteuri* and *S. warneri*.

^6^ Other streptococcal species that were identified include *S. anginosus*, *S. gordonii*, *S. parasanguinis*, *S. pneumoniae* and *S. vestibularis*.

^7^ Other Firmicutes includes *Bacillus*, *Lactococcus*, former *Lactobacillus* and *Weisella*.

^8^ Proteobacteria includes *Brevundimonas, Enterobacteriaceae, Moraxella, Rhizobium* and *Stenotrophomonas*.

IQR: interquartile range; CFU: colony-forming units.

**Supplementary Table 5.** Concentration (nmol/mL) of individual HMOs in milk samples according to mastitis type: acute (AM) or subacute (SAM) mastitis.

| **HMO** | **AM (n = 29)** | **SAM (n = 40)** | ***p-*value^1^** |
| --- | --- | --- | --- |
|  | **Median (IQR) or**  **mean [95% CI]** | **Median (IQR) or**  **mean [95% CI]** |  |
| **Fucosylated** |  |  |  |
| 2'FL | 5924.50 (3902.60-8340.70) | 5921.10 (3313.80-7521.62) | 0.793 |
| 3FL | 1460.40 (1097.75-2941.90) | 2358.00 (1483.12-3644.75) | 0.060 |
| DFLac | 297.90 (235.50-399.60) | 351.15 (247.12-498.65) | 0.317 |
| LNFP I | 1633.70 (630.70-2135.25) | 965.25 (416.27-1510.60) | 0.317 |
| LNFP II | 798.90 (532.20-1443.20) | 781.60 (542.15-1166.67) | 0.806 |
| LNFP III | 24.90 (16.90-39.40) | 14.15 (7.95-24.07) | *0.042 |
| DFLNT | 1120.72 [222.32] | 987.35 [177.29] | 0.800 |
| FLNH | 208.40 (152.30-292.30) | 173.75 (91.87-288.85) | 0.325 |
| DFLNH | 88.30 (41.50-161.70) | 124.00 (57.07-206.62) | 0.325 |
| **Sialylated** |  |  |  |
| 3'SL | 314.80 (225.20-401.90) | 331.15 (251.15-444.22) | 0.542 |
| 6'SL | 800.15 (649.80-934.50) | 618.15 (346.35-823.22) | 0.187 |
| LSTb | 74.80 (48.10-91.30) | 54.20 (39.95-92.70) | 0.334 |
| LSTc | 284.30 (199.50-382.50) | 162.10 (56.40-249.72) | 0.057 |
| DSLNT | 212.50 (107.90-258.60) | 90.45 (28.67-167.05) | *0.032 |
| FDSLNH | 166.75 [31.50] | 134.05 [31.17] | 0.326 |
| DSLNH | 69.10 (47.50-97.70) | 57.45 (31.80-96.32) | 0.472 |
| **Neutral** |  |  |  |
| LNnT | 427.30 (320.80-536.90) | 230.00 (161.42-353.80) | *0.000 |
| LNT | 1510.10 (1235.30-2159.35) | 986.90 (565.45-1436.55) | *0.000 |
| LNH | 55.50 (33.30-112.25) | 58.00 (34.47-95.02) | 0.966 |

^1^Wilcoxon rank sum test or ANOVA tests (depending on the distribution of the data) were used to determine differences in HMOs concentration between samples from AM or SAM cases. FDR-adjusted *p*-values. *Statistically significant difference, *p* < 0.05.

CI, confidence interval; IQR. Interquartile range. DFLac, difucosyllactose; DFLNH, difucosyllacto-*N*-hexaose; DFLNT, difucosyllacto-*N*-tetrose; DSLNH, diasilyllacto-*N*-hexaose; DSLNT, diasilyllacto-*N*-tetraose; FDSLNH, fucodisialyllacto-*N*-hexaose; FLNH, fucosyllacto-*N*-hexaose; HMO, human milk oligosaccharide; LNFP, lacto-*N*-fucopentaose; LNH, lacto-*N*-hexaose; LNnT, lacto-*N*-neotetraose; LNT, lacto-*N*-tetraose; LSTb, sialyl-lacto-*N*-tetraose b; LSTc, sialyl-lacto-*N*-tetraose c; 2’FL, 2’-fucosyllactose; 3FL, 3-fucosyllactose; 3’SL, 3’-sialyllactose; 6’SL, 6’-sialyllactose.

**Supplementary Table 6.** Concentration (nmol/mL) of grouped HMOs in milk samples according to mastitis type: acute (AM) or subacute (SAM) mastitis.

| **HMO** | **AM (n = 29)** | **SAM (n = 40)** | ***p-*value^1^** |
| --- | --- | --- | --- |
|  | **Median (IQR)**  **or mean [95% CI]** | **Median (IQR)**  **or mean [95% CI]** |  |
| HMO-bound fucose acid^2^ | 15146.85 (12164.10-16000.60) | 14379.75 (11790.13-16667.88) | 0.957 |
| HMO-bound sialic acid^3^ | 2409.92 [336.68] | 2034.44 [332.44] | 0.212 |
| Small HMOs^4^ | 9003.99 [954.95] | 9608.76 [806.10] | 0.390 |
| Type 1 HMOs^5^ | 4588.3 [469.97] | 3419.25 [447.15] | *0.000 |
| Type 2 HMOs^6^ | 748.60 (520.70-940.30) | 417.30 (269.55-671.00) | *0.005 |
| α-1,2^7^ | 8038.90 (5273.80-9768.05) | 6998.10 (3959.25-9010.00) | 0.558 |
| α-1,3^8^ | 1508.30 (1161.15-2992.00) | 2372.75 (1530.40-3652.50) | 0.073 |
| α-2,6^9^ | 1015.10 (751.35-1243.25) | 864.65 (364.22-1473.10) | 0.588 |
| Evenness | 0.25 (0.17-0.31) | 0.23 (0.18-0.26) | 0.390 |
| Diversity | 4.81 (3.31-5.92) | 4.36 (3.51-5.00) | 0.390 |
| Total HMOs | 17239.40 (14559.20-18433.80) | 16284.25 (13510.93-18320.40) | 0.390 |

^1^Wilcoxon rank sum test or ANOVA tests (depending on the distribution of the data) were used to determine differences in HMOs concentration between samples from AM or SAM cases. FDR-adjusted *p*-values.

*Statistically significant difference, *p* < 0.05.

^2^Calculated as the sum of all fucose moieties bound to each HMO.

^3^Calculated as the sum of all sialic acid moieties bound to each HMO.

^4^Calculated as 2'FL + 3FL + 3'SL + 6'SL.

^5^Calculated as LNT + LNFP I + LNFP II + LSTb + DSLNT.

^6^Calculated as LNnT + LNFP III + LSTc.

^7^Calculated as LNFP I + 2’FL

^8^Calculated as LNFP III + 3FL.

^9^Calculated as LSTc + 6'SL.

**Supplementary Table 7.** Frequency and concentration of immune factors detected in milk samples from acute mastitis (AM; n = 19) and subacute mastitis (SAM; n= 35) cases.

| **Compound** |  | **AM (n = 19)** |  | **SAM (n = 35)** | ***p*-value^2^** | ***p*-value^3^** |
| --- | --- | --- | --- | --- | --- | --- |
|  | **n (%)^1^** | **Median (IQR)** | **n (%)** | **Median (IQR)** |  |  |
| **Innate immunity** | | | | | |  |
| IL1β (ng/L) | 19 (100) | 15.51 (3.51-33.60) | 32 (91) | 4.23 (1.01-11.51) | *0.779* | 0.382 |
| IL6 (ng/L) | 17 (89) | 12.76 (4.43-48.23) | 25 (71) | 8.44 (1.08-23.16) | *0.779* | 0.404 |
| IL12(p70) (ng/L) | 2 (11) | 1.62 (1.19-2.06) | 6 (17) | 3.04 (1.09-6.07) | *1.000* | 0.644 |
| IFNγ (ng/L) | 19 (100) | 84.09 (13.47-220.99) | 33 (94) | 48.23 (10.59-427.18) | *0.977* | 0.747 |
| TNFα (ng/L) | 19 (100) | 42.04 (24.93-67.45) | 35 (100) | 21.39 (10.57-94.68) | *1.000* | 0.404 |
| **Acquired immunity** | | |  |  |  |  |
| IL2 (ng/L) | 18 (95) | 5.95 (1.30-9.56) | 20 (57) | 14.27 (0.98-35.33) | 0.072 | 0.382 |
| IL4 (ng/L) | 10 (53) | 0.48 (0.26-0.67) | 18 (51) | 1.04 (0.28-2.39) | *1.000* | 0.382 |
| IL10 (ng/L) | 0 (0) | - | 6 (17) | 3.51 (3.29-4.40) | *0.480* | - |
| IL13 (ng/L) | 1 (5) | 0.04 | 9 (25) | 0.76 (0.76-1.24) | *0.480* | 0.404 |
| IL17 (ng/L) | 13 (68) | 17.04 (11.89-19.55) | 18 (51) | 31.11 (8.2-63.56) | 0.779 | 0.404 |
| **Chemokines** | | |  |  |  |  |
| IL8 (ng/L) | 19 (100) | 351.30 (208.98-2140.61) | 35 (100) | 195.12 (90.78-1406.96) | *1.000* | 0.382 |
| MCP1 (ng/L) | 19 (100) | 537.96 (201.35-1080.37) | 35 (100) | 376.36 (58.33-728.20) | *1.000* | 0.443 |
| MIP1β (ng/L) | 19 (100) | 65.59 (19.41-162.03) | 35 (100) | 16.80 (9.55-145.50) | *1.000* | 0.404 |
| **Hematopoyetic factors** | | |  |  |  |  |
| IL5 (ng/L) | 5 (26) | 11.03 (8.28-21.32) | 10 (29) | 49.13 (34.43-75.25) | 1.000 | 0.238 |
| IL7 (ng/L) | 6 (32) | 16.74 (8.61-23.32) | 14 (40) | 25.40 (15.54-137.52) | *0.977* | 0.403 |
| GCSF (ng/L) | 19 (100) | 425.25 (74.01-696.52) | 32 (91) | 117.38 (41.85-688.67) | *0.779* | 0.443 |
| GMCSF (ng/L) | 7 (37) | 1.20 (0.43-3.44) | 12 (34) | 4.61 (2.67-5.10) | 0.954 | 0.382 |
| TGFβ_2_ (µg/L) | 19 (100) | 1.88 (0.90-5.57) | 35 (100) | 1.09 (0.32-6.53) | *1.000* | 0.544 |

^1^n (%): number (percentage) of samples in which the immunological compound was detected (relative frequency of detection).

^2^χ2 or Fisher tests (in italics) were used to determine a possible association between the AM and SAM cases and the immunological compound detected. FDR-adjusted *p*-values.

^3^Wilcoxon rank sum tests were used to determine differences in the concentration detected of each immunological compound between samples from AM and SAM cases. FDR-adjusted *p*-values.

GCSF, granulocyte colony-stimulating factor; GMCSF, granulocyte-macrophage colony-stimulating factor; INFγ, interferon-γ; IL, interleukin; MCP1, macrophage-monocyte chemoattractant protein-1; MIP1β, macrophage inflammatory protein-1β; TGFβ2, transforming growth factor-β2; TNFα, tumor necrosis factor-α.


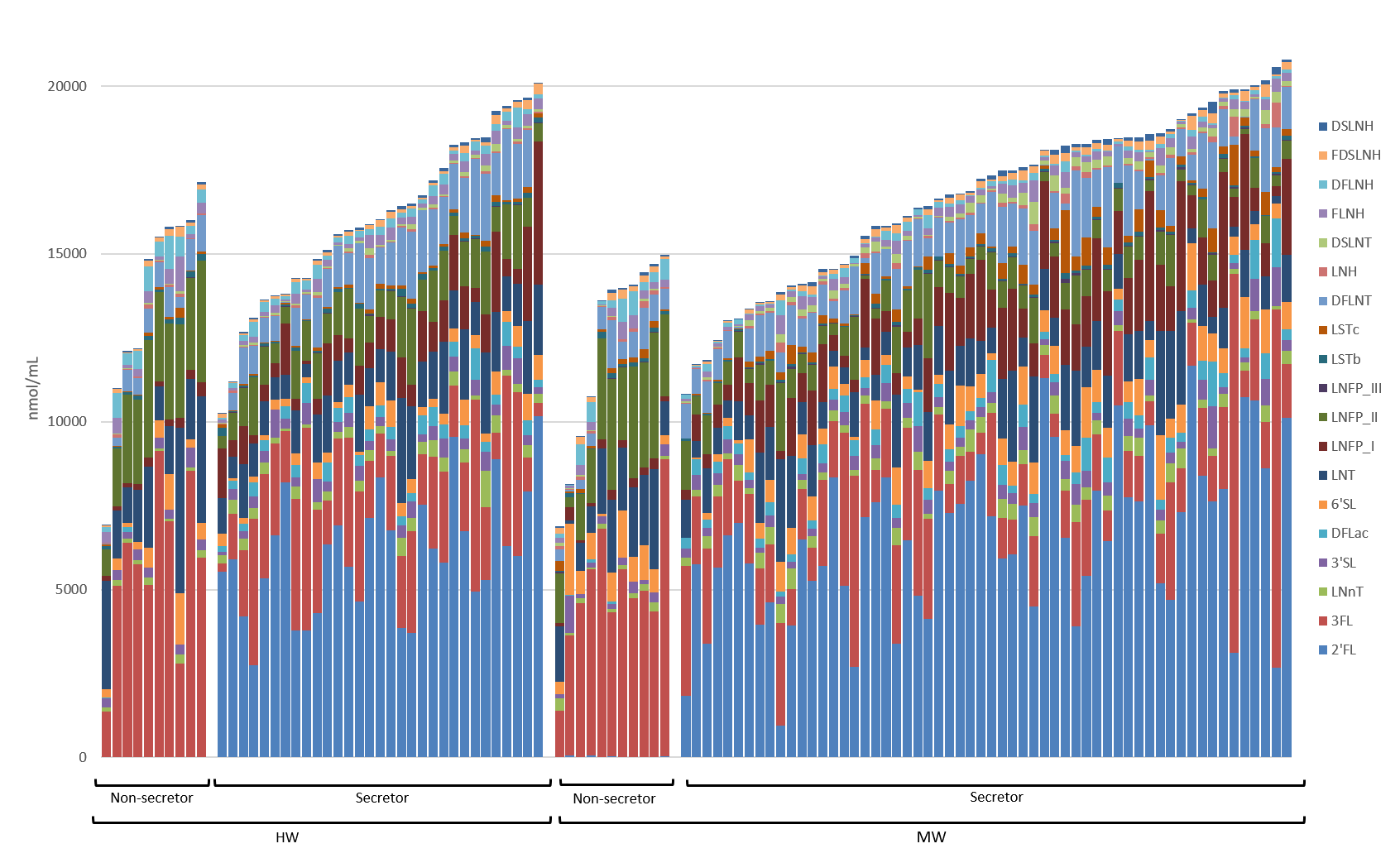


**Supplementary Figure 1**. Absolute HMOs concentration (nmol/mL) and HMO profile in 110 samples from all participant women in the study according to health status (HW, healthy women; MW, mastitis women) and secretory status. Each vertical line represents an individual sample and the concentration of the different HMOs are coded by the colors shown in the legend.

DFLac, difucosyllactose; DFLNH, difucosyllacto-*N*-hexaose; DFLNT, difucosyllacto-*N*-tetrose; DSLNH, diasilyllacto-*N*-hexaose; DSLNT, diasilyllacto-*N*-tetraose; FDSLNH, fucodisialyllacto-*N*-hexaose; FLNH, fucosyllacto-*N*-hexaose; HMO, human milk oligosaccharide; LNFP, lacto-*N*-fucopentaose; LNH, lacto-*N*-hexaose; LNnT, lacto-*N*-neotetraose; LNT, lacto-*N*-tetraose; LSTb, sialyl-lacto-*N*-tetraose b; LSTc, sialyl-lacto-*N*-tetraose c; 2’FL, 2’-fucosyllactose; 3FL, 3-fucosyllactose; 3’SL, 3’-sialyllactose; 6’SL, 6’-sialyllactose.

**
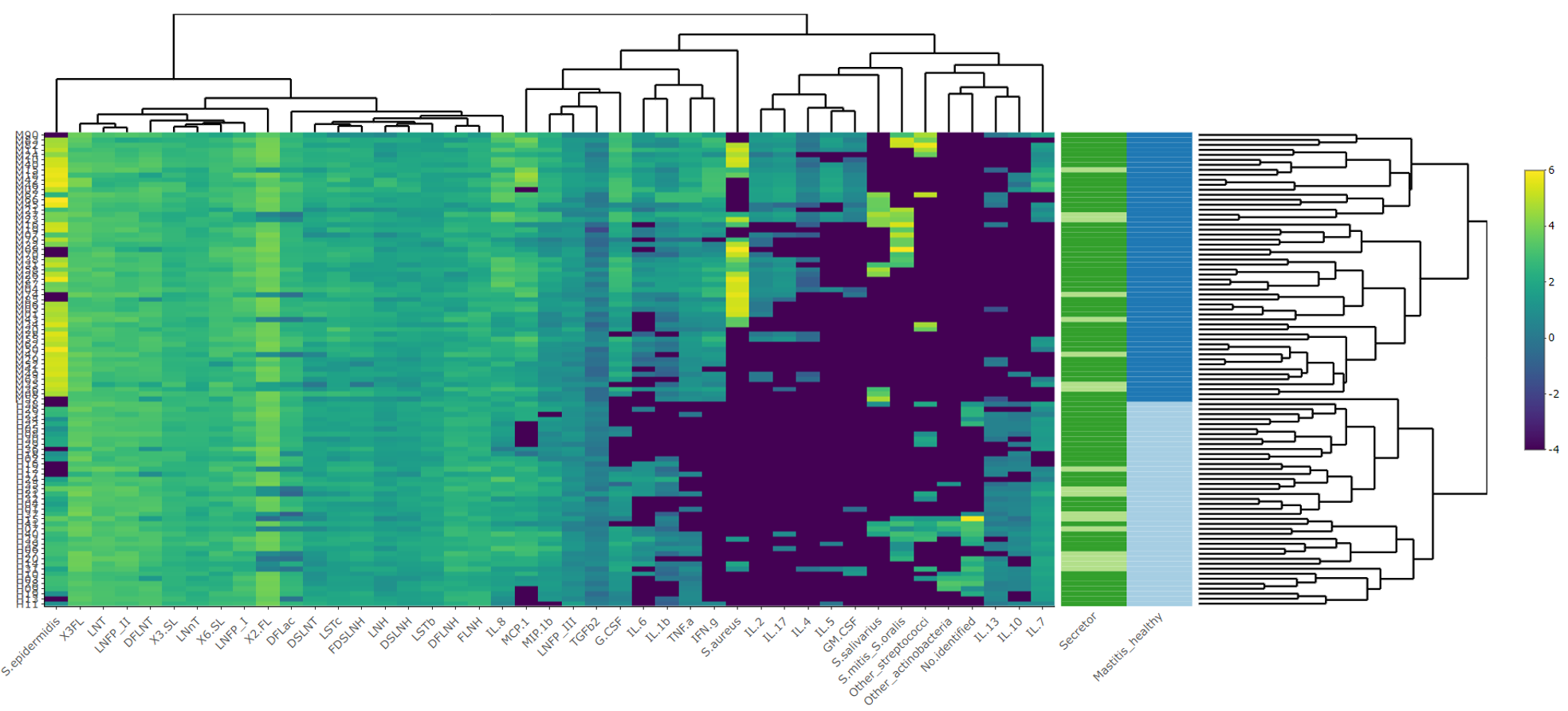
Supplementary Figure 2.** Heatmap showing log_10_-transformed HMO and immunological concentrations and microbiological counts (x axis) in milk samples (y axis; each row represents one milk sample). Samples from women with mastitis (MW group) are color coded in dark blue, and samples from healthy women (HW group) in light blue (Mastitis_Healthy column). Samples from women characterized as Secretors are color coded in dark green and non-Secretors in light green (Secretor column). The concentration of individual or grouped HMOs, immunological compounds and bacterial species within each sample is indicated by the color of the scale ranging from yellow (high concentration) to dark blue (low concentration) as indicated in the scale shown at the right. Upper dendrogram linkages are based on similarities among HMO and immunological compound concentrations and microbial counts; right side dendrogram linkages are based on similarities among the profile of the samples. The heatmap was generated using the *heatmaply* package [1] (v. 1.2.1) available for R (http://cran.r-project.org/package=*heatmaply*).

DFLac, difucosyllactose; DFLNH, difucosyllacto-*N*-hexaose; DFLNT, difucosyllacto-*N*-tetraose; DSLNH, diasilyllacto-*N*-hexaose; DSLNT, diasilyllacto-*N*-tetraose; FDSLNH, fucodisialyllacto-*N*-hexaose; FLNH, fucosyllacto-*N*-hexaose; LNFP, lacto-*N*-fucopentaose; LNH, lacto-*N*-hexaose; LNnT, lacto-*N*-neotetraose; LNT, lacto-*N*-tetraose; LSTb, sialyl-lacto-*N*-tetraose b; LSTc, sialyl-lacto-*N*-tetraose c; 2’FL, 2’-fucosyllactose; 3FL, 3-fucosyllactose; 3’SL, 3’-sialyllactose; 6’SL, 6’-sialyllactose. Other streptococcal species that were identified include *S.* *anginosus*, *S.* *gordonii,* *S. parasanguinis, S. pneumoniae* and *S. vestibularis.* Other Actinobacteria includes *Actinomyces, Bifidobacterium*, *Cutibacterium, Kocuria* and *Propionibacterium.* GCSF, granulocyte colony-stimulating factor; GM.CSF, granulocyte-macrophage colony-stimulating factor; INF.g, interferon γ; IL, interleukin; MCP.1, macrophage-monocyte chemoattractant protein 1; MIP.1b, macrophage inflammatoryprotein 1β; TGFb, transforming growth factor-β_2_; TNF.a, tumor necrosis factor α.

[1] Galili, T., O'Callaghan, A., Sidi, J. & Sievert, C. heatmaply: an R package for creating interactive cluster heatmaps for online publishing. *Bioinformatics*, **34**, 1600-1602; [10.1093/bioinformatics/btx657](https://doi.org/10.1093/bioinformatics/btx657) (2018).


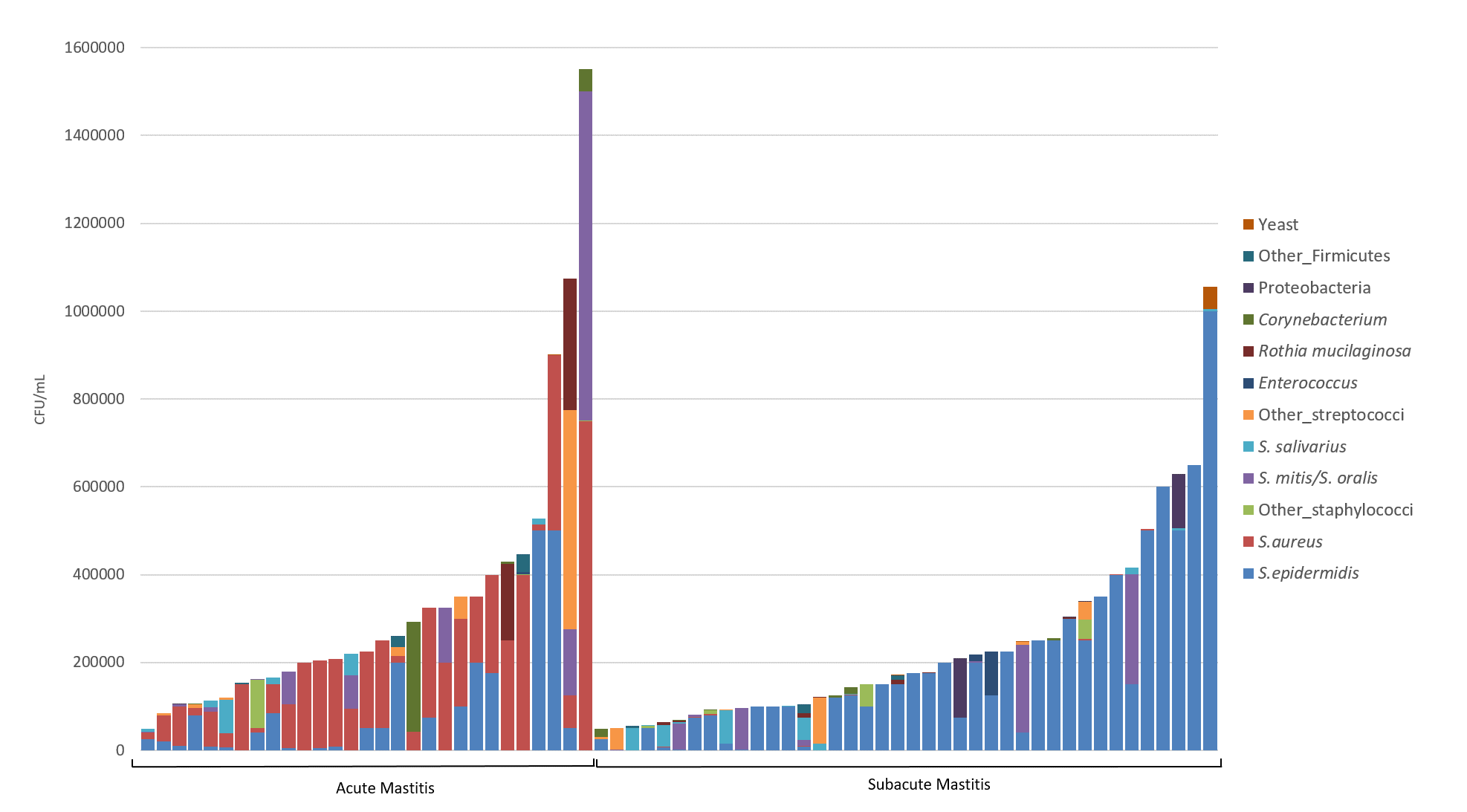


**Supplementary Figure 3**. Microbial profile of samples from acute (AM; n = 29) and subacute mastitis (SAM; n = 40) cases. Each vertical line represents an individual sample and the concentrations (CFU/mL) of individual or grouped microorganism are coded by the colors shown in the legend. Other staphylococcal species that were identified include *S. hominis*, *S. lugdunensis,* *S. pasteuri* and *S. warneri*. Other streptococcal species that were identified include *S. anginosus*, *S. gordonii,* *S. parasanguinis, S. pneumoniae* and *S. vestibularis.* Other Firmicutes includes *Bacillus*, *Lactococcus*, former *Lactobacillus* and *Weisella.* Proteobacteria includes *Brevundimonas*, *Enterobacteriaceae, Moraxella, Rhizobium* and *Stenotrophomonas*.


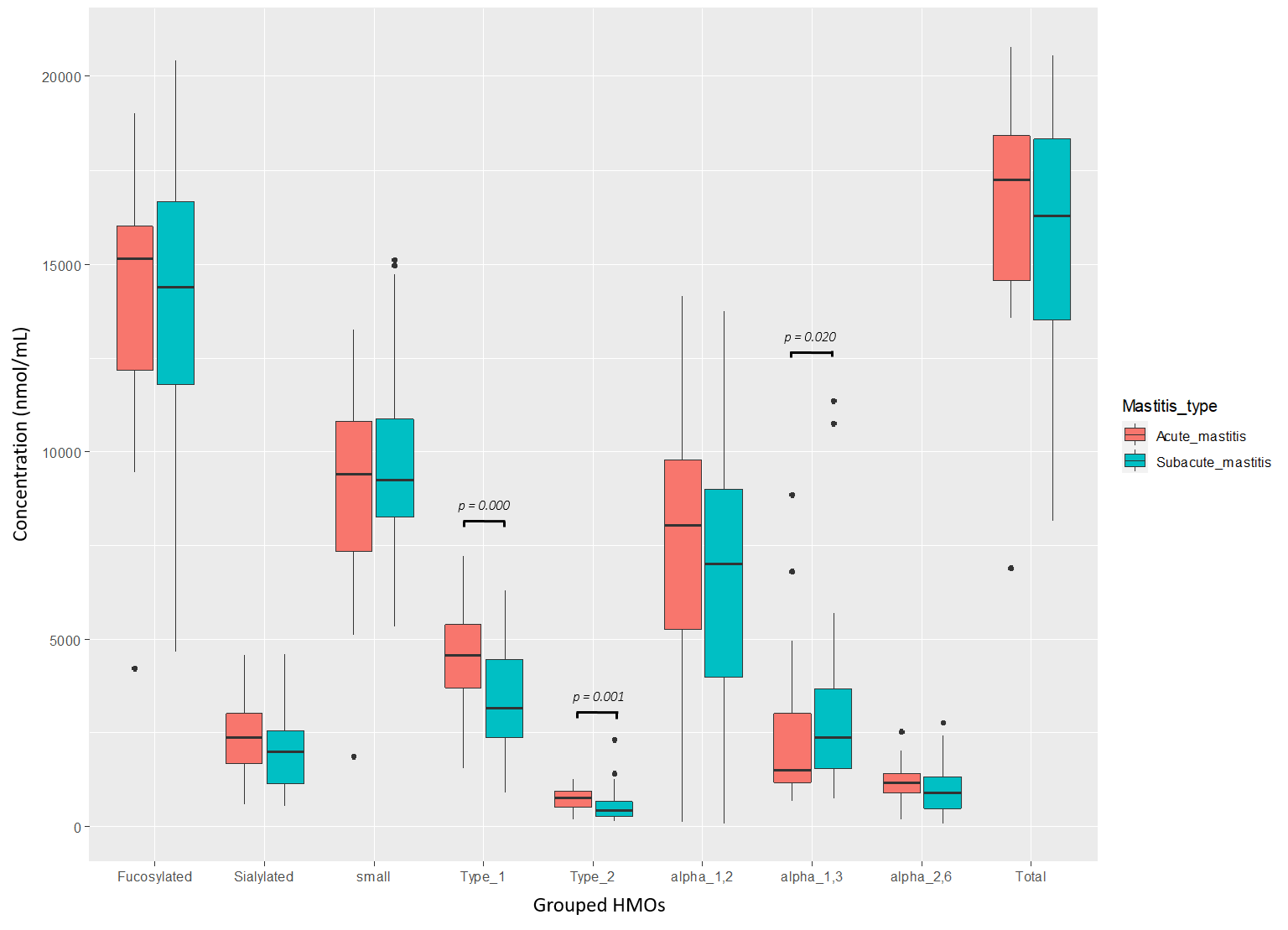


**Supplementary Figure 4.** Box-plots showing the concentrations (nmol/mL) of grouped HMOs. The boxes represent the values of the interquartile ranges, with the median represented as a line. Outliers are represented as dots. Samples colored in red belong to acute mastitis (AM) and in green to subacute m (SAM) cases.

Fucosylated HMOs are calculated as the sum of all sialic acid moieties bound to each HMO; Sialylated HMOs are calculated as the sum of all fucose moieties bound to each HMO; Small HMOs are calculated as 2'FL + 3FL + 3'SL + 6'SL; Type 1 HMOs are calculated as LNT + LNFP I + LNFP II + LSTb + DSLNT; Type 2 HMOs are calculated as LNnT + LNFP III + LSTc; α-1,2 HMOs are calculated as LNFP I + 2’FL; α-1,3 are calculated as LNFP III + 3FL; α-2,6 are alculated as LSTc + 6'SL.

**Supplementary Figure 5.** Receiver operating characteristic (ROC) curve for IL8 concentration in milk used to predict mastitis. The closer the area under the curve (AUC) is to 1.0, the better the IL8 concentration predicts mastitis. Diagnostic accuracy was categorized as failed (ROC AUC ≤ 0.6), poor (0.6 < ROC AUC ≤ 0.7), fair (0.7 < ROC AUC ≤ 0.8), good (0.8 < ROC AUC ≤ 0.9) or excellent (0.9 < ROC AUC ≤ 1.0).
